# Metabolic Profiling of CSF from People Suffering from Sporadic and LRRK2 Parkinson’s Disease: A Pilot Study

**DOI:** 10.3390/cells9112394

**Published:** 2020-10-31

**Authors:** Ali Yilmaz, Zafer Ugur, Ilyas Ustun, Sumeyya Akyol, Ray O. Bahado-Singh, Michael Maddens, Jan O. Aasly, Stewart F. Graham

**Affiliations:** 1 Metabolomics Department, Beaumont Research Institute, Beaumont Health, Royal Oak, MI 48073, USA; zaferugur34@gmail.com (Z.U.); Sumeyya.Akyol@beaumont.org (S.A.); Ray.Bahado-Singh@beaumont.org (R.O.B.-S.); Stewart.Graham@beaumont.org (S.F.G.); 2 Oakland University-William Beaumont School of Medicine, Rochester, MI 48309, USA; Michael.Maddens@beaumont.org; 3 College of Computing and Digital Media, DePaul University, Chicago, IL 60604, USA; IUSTUN@depaul.edu; 4 Department of Internal Medicine, Beaumont Health, Royal Oak, MI 48073, USA; 5 Department of Neurology, St. Olavs Hospital and Department of Neuroscience, Norwegian University of Science and Technology, 7030 Trondheim, Norway; jan.aasly@ntnu.no

**Keywords:** metabolic pathways, Parkinson’s disease, ^1^H NMR, targeted mass spectrometry, metabolomics, machine learning

## Abstract

CSF from unique groups of Parkinson’s disease (PD) patients was biochemically profiled to identify previously unreported metabolic pathways linked to PD pathogenesis, and novel biochemical biomarkers of the disease were characterized. Utilizing both ^1^H NMR and DI-LC-MS/MS we quantitatively profiled CSF from patients with sporadic PD (*n* = 20) and those who are genetically predisposed (LRRK2) to the disease (*n* = 20), and compared those results with age and gender-matched controls (*n* = 20). Further, we systematically evaluated the utility of several machine learning techniques for the diagnosis of PD. ^1^H NMR and mass spectrometry-based metabolomics, in combination with bioinformatic analyses, provided useful information highlighting previously unreported biochemical pathways and CSF-based biomarkers associated with both sporadic PD (sPD) and LRRK2 PD. Results of this metabolomics study further support our group’s previous findings identifying bile acid metabolism as one of the major aberrant biochemical pathways in PD patients. This study demonstrates that a combination of two complimentary techniques can provide a much more holistic view of the CSF metabolome, and by association, the brain metabolome. Future studies for the prediction of those at risk of developing PD should investigate the clinical utility of these CSF-based biomarkers in more accessible biomatrices. Further, it is essential that we determine whether the biochemical pathways highlighted here are recapitulated in the brains of PD patients with the aim of identifying potential therapeutic targets.

## 1. Introduction

Parkinson’s disease (PD) is a progressive, adult-onset neurodegenerative disorder associated with the degeneration of dopaminergic (DAergic) neurons and the presence of proteinaceous inclusions such as α-synuclein [1]. PD is the second most common neurodegenerative disorder after Alzheimer’s disease with a prevalence of 1% in people over 60 years of age [2]. The etiology of PD is thought to be multifunctional in that genetic factors, environmental exposures, and aging contribute to disease development [3]. Genetic studies revealed several causative monogenetic mutations that account for a small portion (10%) of PD cases, while the remaining cases are sporadic [4]. The exact underlying mechanism for selective DAergic cell loss in PD remains elusive [5].

Clinically, the diagnostic criteria for PD are overwhelmingly based on impaired motor and non-motor functions and confirmed following improvements with dopamine treatment [6]. However, the accuracy of such diagnostic criteria is low, as 40% to 50% of DAergic neurons have already degenerated by the time a clinical diagnosis is made [7]. Moreover, premotor symptoms, including olfactory deficiency, obstipation, sleep disorders, and depression are very unspecific to PD, further complicating early diagnosis [8].

The development of molecular biomarkers, specific to PD, in body fluids such as urine, saliva, blood, and CSF, would prove invaluable, allowing detection of the disease at the preclinical stage, monitoring the progression of the disease, and evaluating the therapeutic impacts of disease modifiers. Metabolomics, or metabolic profiling, is the quantitative measurement of metabolites in cells, biofluids, tissues, or organisms, and accurately represents the biochemical phenotype of the organism in diseased and healthy states [9]. In recent years, metabolomics has been widely applied for the study of PD and has shown great potential for early diagnosis [10,11]. CSF is a widely used biomatrix used for developing biomarkers of CNS disorders, such as brain injury [12], Alzheimer’s disease [13], and PD [7], due to its close localization to the extracellular space in the brain. To the authors’ knowledge, this is the first targeted metabolomics study combining two complimentary metabolomics platforms to provide a holistic view of the CSF metabolome, and inversely, the brain metabolome to gain insight into PD pathophysiology and preclinical diagnosis

## 2. Materials and Methods

### 2.1. CSF Samples

The study was approved by the Institutional Review Board at the Beaumont Research Institute and at St. Olav’s Hospital (IRB# 201 2016/541, PD and markers for progression). Written informed consent was obtained from all participants who presented in the morning following an overnight fast with a stable neurological condition. All participants were sequenced for the main pathogenic Leucine-Rich Repeat Kinase 2 (LRRK2) mutations prior to sample collection. A total of 80 subjects with no known history of liver dysfunction were enrolled in this study; CSF specimens were obtained via lumbar puncture from participants suffering from sPD (*n* = 20), those genetically predisposed to the disease (LRRK2 acquired PD) (*n* = 20), and age and gender-matched controls for each group to include patients with the G2019S mutation in the LRRK2 gene (*n* = 20) and those without (*n* = 20). Samples were immediately processed according to the Parkinson’s Progression Markers Initiative (PPMI) recommendations [14] that include removal of cells to avoid any contamination originating from potential blood cells.

### 2.2. ^1^H NMR Analysis

Samples were thawed at room temperature. CSF samples were filtered through 3-kDa cut-off centrifuge filter units (Amicon Micoron YM-3; Sigma-Aldrich, St. Louis, MO, USA) to remove any protein. Centrifugal filters were washed 7 times prior to filtering CSF specimens to remove excess glycerol [15] An aliquot of 350 µL of each CSF sample was filtered at 13,000× *g* for 30 min to remove large macromolecules. The samples were prepared by transferring a 300 μL aliquot of filtered CSF fluid to a 1.5 mL Eppendorf tube followed by the addition of 35 μL of D_2_O, and 15 μL of a standard solution (3.73 mM disodium-2,2-dimethyl-2-silapentane-5-sulphonate [DSS]), 233mM imidazole, and 0.47% NaN_3_ in H_2_O (Sigma-Aldrich, St. Louis, MO, USA). The CSF sample (200 μL) was then transferred to a standard 3 mm NMR tube. In total, 80 CSF samples were prepared in this manner, each containing 0.16 mM DSS, 10 mM imidazole, and 0.02% NaN_3_ at a pH of 7.3 to 7.7. All ^1^H NMR spectral data were acquired at 300 (± 0.05) K using a Bruker Ascend III HD NMR spectrometer (Bruker-Biospin, Billerica, MA, USA) operating at 600 MHz equipped with a 5 mm TCI cryo-probe and a z-gradient system; a Bruker SampleJet sample changer was used to transfer samples; samples were kept at 4 °C while queued for analysis. Prior to analysis, samples were heated to room temperature over 3 min before being transferred to the magnet. ^1^H NMR spectra were acquired at 300 K using the first transient of a standard NOESY-presaturation pulse sequence, chosen for its high degree of high quantitative accuracy [15]. The duration of the 90-degree pulses was automatically calibrated for each individual sample using a homonuclear gated nutation experiment on the locked and shimmed samples after automatic tuning and matching of the probe head. Spectra were collected with 256 transients using a 5.1 s acquisition time and a 5 s recycle delay. Prior to spectral analysis, all FIDs were zero-filled to 64k data points, and a line broadening of 0.5 Hz was applied. The methyl singlet of DSS served as an internal standard for chemical shift referencing (set to 0 ppm) and for quantification. All ^1^H NMR spectra were processed and analyzed using the Chenomx NMR Suite Professional software package version 8.1 (Chenomx Inc., Edmonton, AB, Canada). Prior to statistical analysis, all NMR spectra were manually inspected for technical faults.

### 2.3. Targeted Mass Spectrometry Analysis

Using the commercially available AbsoluteIDQ p180 Kit (Biocrates Life Sciences AG) [16] we biochemically profiled the CSF specimens. The samples were processed according to the manufacturer’s instructions. In brief, 30 μL of each CSF sample was mixed with isotopically labeled internal standards in a 96-well plate. Amino acids and biogenic amines were derivatized using 5% phenylisothiocyanate (PITC), and subsequent separation was performed on a BEH C18 column (2.1 × 75 mm, particle size of 1.7 μm) on a Waters iClass coupled to a Waters Xevo TQ-S (Waters, Milford, MA, USA) operating in the multiple reaction mode. The subsequent lipid fraction was analyzed using direct flow, operating in multiple reaction monitoring mode. Metabolite concentrations were calculated and expressed as μM. Any metabolites below the limit of detection (LOD) were excluded from our analysis.

### 2.4. Bile-Acid Analysis

Bile acids were analyzed using the commercially available Biocrates Bile Acids Kit (Biocrates Life Science AG, Innsbruck, Austria) as described by Marksteiner et al. [16]. This assay allows identification and quantification of primary and secondary bile acids and their taurine and glycine conjugated derivatives. The metabolite panel included 17 individual bile acids, corresponding internal standards, and calibration ranges. Data analysis was performed with TargetLynx (Waters) and Biocrates MetIDQ software.

### 2.5. Statistical Analysis

#### 2.5.1. Univariate Data Analysis

Using MetaboAnalyst (v. 4.0) [17,18,19], the data were analyzed using a two-tailed Student *t*-test and ANOVA to determine statistical significance (*p*-value) and false discovery rates (FDR; *q*-value). To assess the distribution of our data and to check for any spurious/inflated results, quantile-quantile plots (Q–Q-plots) were generated. For non-normal distributions, *p*-values were calculated using a Mann–Whitney U test. Bonferroni corrected *p*-values (*p* = 0.05/number of metabolites) were used to correct for multiple comparisons.

#### 2.5.2. Multivariate Data Analysis

To ensure no violation of the normality assumption, data were standardized by sum normalization, followed by scaling to mean zero and unit variance, and log transformed. Before performing pattern recognition, metabolomics data from each individual group were subjected to explorative multivariate statistical data analysis by PCA to check for potential outliers or systematic variation (*p* < 0.05). This was further investigated by plotting T^2^-values (distance to model) against residual Q2 (the variance not covered by the model) for each group. Following outlier detection, orthogonal projections to latent structure discriminant analysis (O-PLS-DA) was employed to determine if it would be possible to differentiate LRRK2 PD from the LRRK2 control, sPD from the sPD control, and LRRK2 PD from sporadic PD, and to identify features responsible for the observed separation. For each model, the optimum number of components was assessed by a single 10-fold venetian blind cross-validation. The validity of the group separation for each model was evaluated through permutation testing with 2000 repeats. The raw metabolomics data were also subjected to unsupervised hierarchical clustering modelling to generate heat map representation between each group.

#### 2.5.3. Machine Learning-Based Regression Analysis

Before starting an intensive investigation on the predictive performance of various machine learning algorithms, missing values in the dataset were imputed by using a KNN algorithm. Sum normalized data were subjected to log transformation and auto-scaling was applied for each metabolite. A recursive feature elimination (RFE) method using logistic regression was applied to each pair-wise comparison (LRRK2 control vs. LRRK2 PD, sPD control vs. SPD, and LRRK2 PD vs. sPD) to find the most discriminative variables [20,21,22]. Feature selection using the RFE method and subsequent model training were performed using 60% of the data. The remaining 40%, which were excluded from both the feature selection and model training phases, were used to test the models. We used the top 5 discriminant variables to avoid overfitting. Following variable selection, numerous machine learning models were built. To find the optimal combination for each algorithm, hyper-parameters such as cost values, kernel functions, and the numbers of trees to train the data set were tuned; 10-fold cross validation was performed during model development. Once an optimal model was developed, prediction was carried out through 5-fold cross validation. Using optimal hyperparameters, models were trained on 4 folds and tested on the remaining fold. This was repeated 5 times across all datasets. In small datasets, one potential pitfall is that randomly splitting the data into training and test sets may bias the models, giving overly optimistic results. In order to prevent this, 5-fold cross validations were used for prediction.

The synthetic minority over-sampling technique (SMOTE) method from the imbalanced-learn library in python and KNN were used to account for oversampling. Different number of neighbors and different number of data points for oversampling were tested to determine the best combination. Using the k-fold approach, only the training portion was oversampled, and the test set was kept original. Model performance was assessed by classification accuracy rate, using the area under the receiver operating characteristic (AUROC) curve, and by the identification of true discriminating features.

#### 2.5.4. Metabolite Pathway Enrichment Analysis

Metabolite pathway enrichment analysis (MSEA) was completed using MetaboAnalyst (v4.0) [23]. Metabolite names were converted to Human Metabolite Database (HMDB) identifiers, and the raw data were imported in rows, normalized to the sum, and subjected to log transformation and auto scaling. The pathway-associated metabolite set was the chosen metabolite library, and all compounds in this library were used. Pathways with a Holm corrected *p* value and a *q* value < 0.1 were considered altered due to parkinsonism.

## 3. Results

### 3.1. Statistical and Metabolite Pathway Enrichment Analysis

Using both ^1^H NMR (Supplementary Figure S1) and targeted mass spectrometry we accurately identified and quantified 162 metabolites in CSF to include an additional 12 bile acids. Of the recorded metabolites, 17 were measured across both platforms and an average concentration was used. As shown in Supplementary Figure S2, for each group, the PCA score plots and T^2^ vs. Q^2^ plots clearly demonstrate that no patient sample was considered to be an outlier. The Q–Q plots and corresponding λ-values (a measure of inflated *p*-values) reveal that the majority of data points fell within the expected range (λ **≈** 1). Further, our analyses identified some non-normally distributed metabolites which require their statistical significance to be calculated using non-parametric testing (Supplementary Figure S3). Table 1 reports the results of multi-group comparisons of important demographic factors, such as age and gender. The results of the ANOVA revealed that neither gender nor age were statistically significantly different between the groups.

Supplementary Figure S4 displays the box plot representative of all the metabolites detected and quantified in CSF. Using these metabolite concentrations, four pair-wise univariate analysis were carried out (sporadic PD (sPD) vs. sPD controls, LRRK2 PD vs. LRRK2 controls, sPD vs. LRRK2 PD, and sPD controls vs. LRRK2 controls).

Supplementary Table S1 lists the results of the univariate analysis, thereby comparing the mean concentrations of all metabolites from LRRK2 PD vs. LRRK2 control participants. As shown in the table, of the recorded metabolites, 18 metabolites reached statistical significance (*p* < 0.05; *q* < 0.1). We also identified six biochemical pathways to be significantly perturbed in LRRK2 PD CSF as compared with LRRK2 controls (*p* < 0.05; *q* < 0.1; Supplementary Table S2) and these include: spermidine and spermine biosynthesis, fatty acid metabolism, mitochondrial beta oxidation of long chain fatty acids (LCFAs), methionine metabolism, bile-acid metabolism, methionine metabolism, and beta oxidation of short chain fatty acids (SCFAs). Supplementary Table S3 lists the results of the univariate analysis, comparing the mean concentrations of all metabolites from sPD vs. sPD control participants. As shown in the table, of the recorded metabolites, 19 metabolites reached statistical significance (*p* < 0.05; *q* < 0.1). Following the univariate analysis, pathway enrichment analysis highlighted five metabolic pathways to be significantly disturbed in sporadic PD as compared to their controls. These include fatty acid biosynthesis, ethanol degradation, ketone body metabolism, bile acid synthesis, and propionate metabolism (*p* < 0.05; *q* < 0.1; Supplementary Table S4).

The univariate analysis comparing the mean concentrations of all metabolites from sPD vs. LRRK2 PD participants revealed that 10 metabolites showed statistically significant concentration changes between the two groups (*p* < 0.05; *q* < 0.1; Supplementary Table S5). MSEA also showed that metabolic pathways, namely, propionate metabolism, inositol metabolism, and inositol phosphate metabolism, were significantly disturbed when sPD was compared with LRRK2 PD CSF (*p* < 0.05; *q* < 0.1; Supplementary Table S6). The results of the pair-wise comparison of PD controls vs. LRRK2 controls indicated that none of the metabolites were at statistically significant different concentrations (Supplementary Table S7).

**Figure 1:**
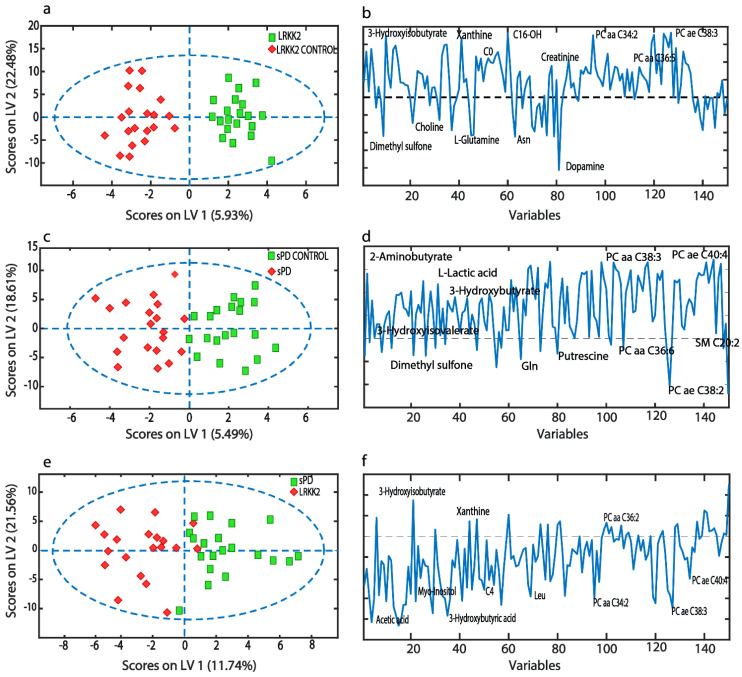
(**a**) The O-PLS-DA score plot of LRKK control vs. LRRK2 sufferers; (**b**) top 13 metabolites on loadings driving the separation; (**c**) O-PLS-DA score plot of sporadic Parkinson’s disease (sPD) controls vs. sPD sufferers; (**d**) top 13 metabolites on loadings driving the separation; (**e**) the O-PLS-DA score plot of sPD vs. LRKK2 sufferers; (**f**) top 13 metabolites on loadings driving the separation.

Figure 1a,c illustrates the O-PLS-DA) score plots for all the pair-wise comparison models and corresponding loading plots driving the separation. As evidenced from the score plots, classification of each group was successfully achieved. However, following 2000 permutations (Supplementary Table S8) only the score plot comparing LRRK2 PD with corresponding controls reached statistical significance (*p* = 0.032).

### 3.2. Machine Learning-Based Classification Analysis

**Figure 2:**
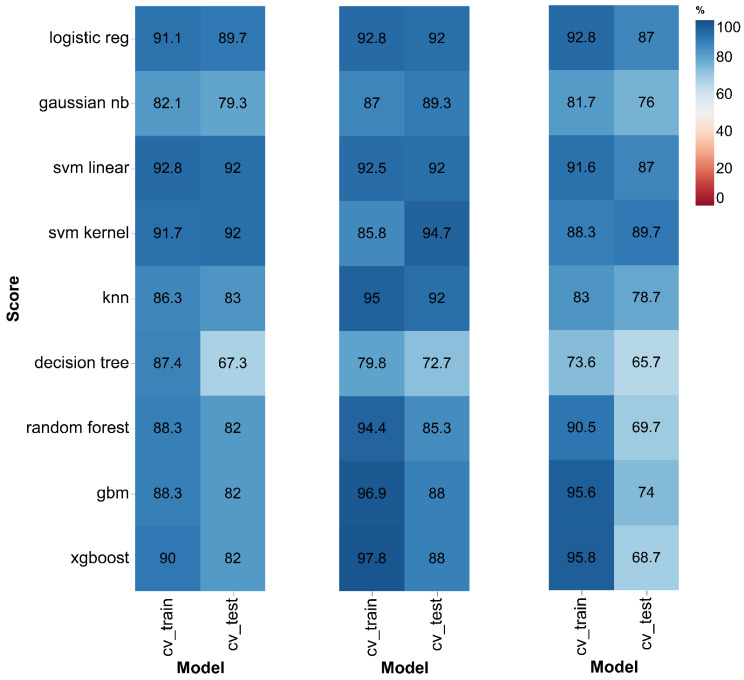
AUC values of various machine learning algorithms evaluated for the prediction of LRRK2 PD as compared LRRK2 controls, sPD as compared to sPD controls, and LRRK2 PD as compared to sPD on both test and training sets, respectively.

The utility of different machine learning approaches for diagnosing parkinsonism was intensively evaluated. Performance metrics, model specific tuning parameters, and the panel of metabolites used for each approach are listed in Supplementary Tables S8–S10. When comparing the traditional multivariate approach with machine learning, we found the latter to works best for diagnosing PD. In particular, we found logistic regression, random linear support vector machine algorithms, and kernel support vector machine algorithms produced the best diagnostic accuracy (Figure 2).

## 4. Discussion

To the authors’ knowledge, this is the first reported study to identify sPD and LRRK2 PD-specific metabolic signatures in CSF using a targeted metabolomics approach employing both ^1^H NMR spectroscopy and mass spectrometry. Using complementary techniques allowed us to detect and quantify many more metabolites, and as such we required much more robust and accurate analytical data tools to help us discriminate between sample types. Further, we compared our values recorded herein with existing metabolite CSF concentration values in the literature [24]. Wishart et al. (2008) reported a variation of 20–30% between all metabolites in CSF between various studies. Our data conform to these values, as reported in the literature [25].

The primary objective of this study was to identify a panel of biomarkers which could accurately diagnose PD using CSF and to gain an insight into the biochemistry behind PD. To ensure we got the most accurate snapshot of the disease’s metabolome, only samples from unmedicated participants were utilized to minimize any confounding factors.

When diagnosing LRRK2 PD, logistic regression (AUC = 0.89), linear support vector machine (SVM) (AUC = 0.92), and SVM kernel (AUC = 0.92) performed similarly and outscored the other ML-based algorithms (Supplementary Figure S5). For distinguishing sPD from their corresponding controls, logistic regression (AUC = 0.92), linear SVM (AUC = 0.92), and kernel SVM (AUC = 0.94) performed best (Supplementary Figure S6). Kernel SVM (AUC = 0.89), linear SVM (AUC = 0.87), and logistic regression (AUC = 0.87) approaches were superior when distinguishing between sPD and LRRK2 PD (Supplementary Figure S7). In a recent study, Stoessel et al. (2017) investigated PD-specific metabolic changes in CSF using non-targeted mass spectrometry and identified a panel of biomarkers for the diagnosis of PD [24]. Using random forest and PLS-DA, they reported models with AUCs of 0.74 and 0.73, respectively. Goldstein et al. (2012) reported models using biomarker candidates in CSF with 0.89 sensitivity and 0.80 specificity [26]. In another study, Hong et al. (2010) reported a predictive model using DJ-1 and α-synuclein levels in CSF provided with a predictive accuracy equal to 0.77, a sensitivity of 0.94, and a specificity of 0.50 [27]. In a recent study by Mondello et al. (2014), α-synuclein and ubiquitin carboxy-terminal hydrolase L1 (UCH-L1) levels in CSF were used to discriminate PD from controls. Both biomarkers discriminated PD from controls well with an AUC of 0.82, a sensitivity of 0.87, and a specificity of 0.79 [28]. In our study we go one step further. We profiled sporadic and LRRK2 positive patients and produced powerful models with an AUC of 0.88 (sensitivity = 1; specificity = 0.75) for diagnosing sPD and a model with an AUC = 0.94 (sensitivity = 0.88; specificity = 1) for diagnosing LRRK2 PD patients (Supplementary Table S8). To the authors’ knowledge these are some of the most robust diagnostic models in the current literature.

Of note, when we ran a comprehensive pathway analysis, we had to be sure that the observed differences in the metabolic profiles were in fact due to the disease. As such, we compared the metabolic profiles from both control groups and found no metabolites to be at statistically significant different concentrations (*p* < 0.05; *q* < 0.1). MSEA also highlighted several metabolic pathways perturbed in the CSF harvested from LRRK2 PD patients (Supplementary Table S2). One such pathway was fatty acid metabolism. Perturbation in fatty acid metabolism in PD has also been previously reported [29]. An enzymatic deficiency in either fatty acid breakdown or disturbance of fatty acid transport across the mitochondrial membrane due to defects in the carnitine transport system results in dysregulation of fatty acid [30]. Supported by the significant change in the level of carnitines in CSF, we hypothesize that lipid metabolism is directly perturbed as a result of the change in carnitine levels. Brain acylcarnitines support lipid biosynthesis and the activity of antioxidants; they also enhance cholinergic neurotransmission [31]. Further, when oxygen levels are low, the brain transitions from glucose metabolism to anaerobic respiration. Alternatively, it can also shift from glucose metabolism entirely, using fatty acids or ketones during pathological conditions such as neurodegeneration, hypoxia/ischemia, or post-traumatic brain injury [32]. Therefore, the perturbation in fatty acid metabolism we observed could be a potential attempt at attenuating neuronal cell death, further supported by a significant change in the level of 1-methylhistamine which is a metabolite in histamine metabolism. Histamine is a neurotransmitter which is widely distributed throughout the human brain, and an increase in it has been reported to be involved in the histaminergic system in PD [33]. Notably, both mitochondrial beta oxidation of LCFA and mitochondrial beta oxidation of SCFA were significantly disturbed in LRRK2 PD. Taken together, these results suggest a profound change in energy metabolism.

Consistent with an essential role in cellular function, lack of inositol in cells leads to a rapid loss of viability [34]. As inositol metabolic pathway was found to be perturbed, the association between the LRRK2 gene and inositol metabolism needs to be further elucidated.

**Figure 3:**
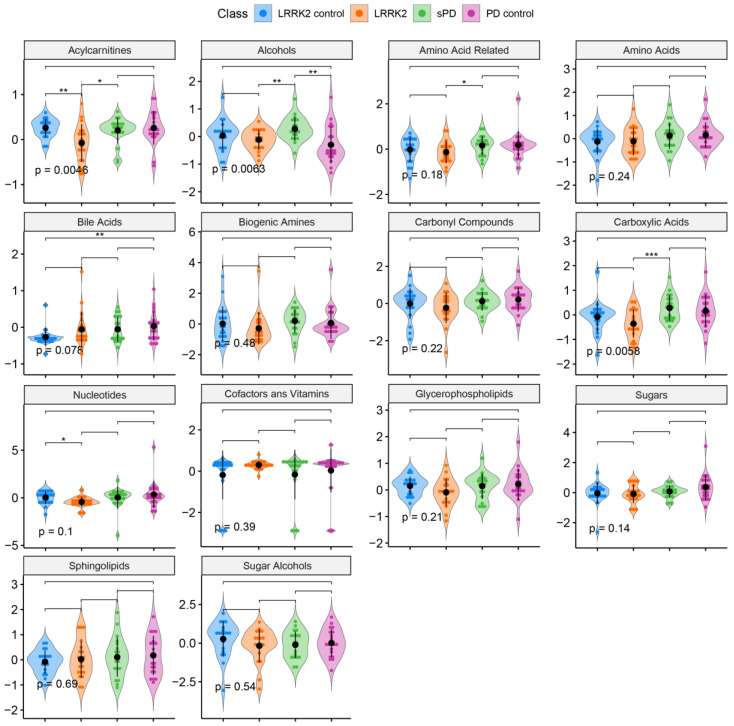
Violin plots comparing the median concentrations of the different metabolite species as identified in the CSF of sPD (*n* = 20), sPD controls (*n* = 20), LRRK2 PD (*n* = 20), and LRRK2 controls (*n* = 20). The data were analyzed using a Student’s *t*-test where ns: *p* > 0.05, *: *p* ≤ 0.05, **: *p* ≤ 0.01, ***: *p* ≤ 0.001. A point in one of the violin plots is representative of a patient sample. Each *p*-value shown on a plot is an ANOVA *p*-value for all the patient groups.

As is evident from the violin plots, we can see that there are dysregulations in several metabolite groups due to parkinsonism (Figure 3).

Close examination of the heat map revealed perturbations in the carnitine, glycerophospholipid, sphingolipid, and amino acid metabolism in PD. MSEA further identified a marked alteration in bile acid metabolism in sPD (Supplementary Table S4). Bile acids are forms of cholinic acids synthesized from cholesterol in the liver [35]. They are involved in many essential biological and metabolic cascades, including glucose, lipid, cholesterol, and drug metabolism, and closely associated with intestinal hormones, microbiotas, and energy balance [36]. However, very little is known about the molecular mechanisms of bile acids in the central nervous system [35]. Previously, changes in bile acid concentrations have been associated with liver damage/dysfunction for some time [37,38]. Following a screening of their medical records and after speaking with the PI of the study, our patients had no reported incidence of liver problems. Changes in bile acid concentrations in PD also validate previous studies by our group, wherein we reported significant changes in bile acid concentrations in both the brain and the serum of a prodromal mouse model of PD [1,39]. A recent study by Bustos et al. (2020), who performed a “hypothesis-free,” exome-wide, burden-based analysis of different variant frequencies, predicted the functional impacts and age of onset classes, in an attempt to explain rare variants in PD. Bustos et al. (2020) report that the top enriched hallmark pathway was for nonsynonymous within the bile acid metabolism M5948, P = 1.63 × 10^−8^ [40].

Another important biochemical pathway found to be aberrant in the CSF of PD patients was taurine and hypotaurine metabolism. Taurine is a major intracellular free β-amino acid in mammalian tissues and intervenes in many physiological functions, such as neuromodulation, maintenance of calcium homeostasis, and antioxidant and anti-inflammatory processes. The level of taurine has been reported to be elevated in the region of the brain controlling the dopamine release and dopaminergic neuron activity [41]. Moreover, taurine has been reported to reduce dopaminergic neurodegeneration and α-synuclein oligomerization through suppression of microglial M1 polarization via NOX2-NF-κB pathway in a pesticide-induced PD model [42]. Thus, one may hypothesize that the perturbation of this particular metabolic pathway could be a neuroprotective reaction by the brain.

It is also noteworthy that this is the first metabolomics study to report ethanol degradation to be significantly perturbed in the CSF of PD patients. Interestingly, one of the key enzymes involved in the ethanol degradation pathway is alcohol dehydrogenase (ADH). Mutations in ADH genes could play a role in the etiology of Parkinson’s disease (PD) because of the important function they undertake, particularly in retinoid and dopamine metabolism and/or aldehyde detoxification [43]. In support of our hypothesis, Tan et al. (2001) reported that a polymorphism at allele A1 for ADH was correlated with an increased risk of PD [44].

Interestingly, propionate metabolism was found to be significantly disturbed between sPD and corresponding controls. (Supplementary Table S4). Propionate, the end-product of the microbial digestion of carbohydrates, presents together with other SCFA in the gastro-intestinal tract. Gut microbiota and their metabolic products are among the potential candidates that could ignite a process that eventually leads to Lewy body formation in the enteric nervous system. In recent studies, differences in the abundances of certain gut microbiota and their metabolic products such as SCFA and propionate were reported [45].

We also report inositol-related metabolic pathways to be perturbed when LRRK2 PD CSF was compared to sPD (Supplementary Table S6). Inositol is an essential metabolite that plays a fundamental role in regulating cellular processes. Many of inositol phosphates and phosphoinositides serve as signaling molecules that control membrane trafficking [46], calcium mobilization [47], chemotaxis [48], ion channel activity [49], cytoskeletal organization [50], and gene expression [51]. Furthermore, phosphatidylinositol is a precursor for the synthesis of sphingolipids, which play important roles in signal transduction and trafficking [52]. Consistent with an essential role in cellular function, lack of inositol in cells leads to a rapid loss of viability [34]. As several inositol related metabolic pathways were perturbed, the association between LRRK2 gene and inositol metabolism needs to be further elucidated.

Our study is not without its limitations. Firstly, our small sample size limits what we can deduce from the results. Secondly, the available clinical information is lacking, such as UPDSR. This precluded us from considering key clinical variables in the models to optimize performance and to determine whether there are other confounding factors that could affect the metabolic profile.

## 5. Conclusions

This study introduces a novel approach to identify potential metabolic pathways and unique biochemical profiles associated with PD pathogenesis using CSF samples. Whereas conventional approaches utilize only one multivariate statistical method, here, the utility and predictive performances of several machine learning methods were investigated. The machine learning predictive model provided highly discriminating models for distinguishing LRRK2 PD from LRRK2 controls, and sPD from sPD controls, respectively. Results suggest, for the first time, that inositol-related metabolic pathways might be the key for better understanding the LRRK2 PD. Future studies for the prediction and diagnosis of those at risk of developing PD should investigate whether the biochemical pathways highlighted here are recapitulated in the brain of PD patients and whether the CSF panel of biomarkers identified are also as effective for diagnosing or predicting those at greatest risk of developing PD in less invasively harvested biomatrices.

## Figures and Tables

**Table 1:** Multi-group comparison of available demographic information.

	sPD Control	sPD	LRRK2 Control	LRRK2 PD	*p*-Value
**n**	20	20	20	20	
**Age, mean (SD)**	57.65 (9.56)	58.85 (8.95)	60.05 (9.39)	59.43 (9.11)	0.45 ^a^
**Gender**					
Male	10	11	10	10	0.26 ^b^
Female	10	9	10	10	

^a^ One-way ANOVA. ^b^ Chi-square test.

## Data Availability

Supporting tables and figures can be reached in supplementary materials. Metabolomics data have been deposited to the EMBL-EBI MetaboLights database with the identifier MTBLS863. The complete dataset can be accessed here: https://www.ebi.ac.uk/metabolights/MTBLS863.

## Supplementary Materials

The following are available online at https://www.mdpi.com/2073-4409/9/11/2394/s1, Figure S1: Representative 1D 1H NMR spectrum of CSF; identified and quantified metabolites are displayed, Figure S2: Investigation of outliers for each group by PCA (a-d) and Q2 vs. T2Hotelling plots(e-f), Figure S3: QQ-plots used to determine whether the metabolomics data for each metabolite are normally distributed and/or the corresponding p-value is inflated, Figure S4: Box-plots showing pair-wise comparisons of the concentration values of all metabolites in CSF, Figure S5: AUROC plot for the top three ML predictive models for the classification of LRRK2 PD as compared to their corresponding controls, Figure S6: AUROC plot for the top three ML based predictive models for the classification of sPD as compared to their corresponding controls, Figure S7: AUROC plot for the top three ML based predictive models for classification of sPD as compared to LRRK2 PD. Table S1: Results of univariate analysis comparing the mean concentration of metabolites in CSF obtained from LRRK2 control and LRRK2 PD patients, Table S2: The results of the Metabolite Set Enrichment Analysis showing the metabolic pathways that were significantly perturbed when LRRK2 PD CSF was compared with their respective controls, Table S3: Results of univariate analysis comparing the mean concentration of metabolites in the CSF obtained from LRRK2 control and LRRK2 PD patients, Table S4: The results of the Metabolite Set Enrichment Analysis showing the metabolic pathways that were significantly perturbed when sPD CSF was compared with their respective controls, Table S5: Results of univariate analysis comparing the mean concentration of metabolites in CSF obtained from sPD and LRRK2 PD patients, Table S6: The results of the Metabolite Set Enrichment analysis showing the metabolic pathways that were significantly perturbed when LRKK2 PD CSF was compared with sPD, Table S7: Results of univariate analysis comparing the mean concentration of metabolites in CSF obtained from sPD controls and LRRK2 PD controls, Table S8: Pairwise O-PLS-DA model performance metrics, Table S9: Optimized model parameters for each machine learning algorithm evaluated for prediction of sPD and LRRK2 PD in this study, Table S10: The panel of metabolites as chosen using the RFE feature selection algorithm to generate our predictive models.
