# Supplementary material for: Metabolic Profiling of CSF from People Suffering from Sporadic and LRRK2 Parkinson’s Disease: A Pilot Study
Source: Cells. 2020 Oct 31;9(11):2394. doi: 10.3390/cells9112394 (PMC7693941; doi:10.3390/cells9112394)

Metabolic Profiling of CSF from People Suffering from Sporadic and LRRK2 Parkinson's Disease: A Pilot Study

Ali Yilmaz [1,2,*], Zafer Ugur [1], Ilyas Ustun [3], Sumeyya Akyol [1], Ray O. Bahado-Singh [1,2], Michael Maddens [2,4], Jan O. Aasly [5] and Stewart F. Graham [1,2]

[1] Metabolomics Department, Beaumont Research Institute, Beaumont Health, Royal Oak, MI 48073, USA; zaferugur34@gmail.com (Z.U.); Sumeyya.Akyol@beaumont.org (S.A.); Ray.Bahado-Singh@beaumont.org (R.O.B.-S.); Stewart.Graham@beaumont.org (S.F.G.)

[2] Oakland University-William Beaumont School of Medicine, Rochester, MI 48309, USA; Michael.Maddens@beaumont.org

[3] College of Computing and Digital Media, DePaul University, Chicago, IL 60604, USA; IUSTUN@depaul.edu

[4] Department of Internal Medicine, Beaumont Health, Royal Oak, MI 48073, USA

[5] Department of Neurology, St. Olavs Hospital and Department of Neuroscience, Norwegian University of Science and Technology, Trondheim, 7030, Norway; jan.aasly@ntnu.no

* Correspondence: ali.yilmaz@beaumont.org; Tel: +1-248-551-2377

Received: 21 August 2020; Accepted: 20 October 2020; Published: date

Search Terms: Parkinson Disease, ^{1}H NMR, targeted Mass spectrometry, metabolomics, Machine learning

Page Number	Table of Contents
P3-8	**Table S1:** Results of univariate analysis comparing the mean concentrations of metabolites in CSF obtained from LRRK2 control and LRRK2 PD patients.
P9	**Table S2:** The results of the Metabolite Set Enrichment Analysis showing the metabolic pathways that were significantly perturbed when LRRK2 PD CSF was compared with their respective controls.
P10-15	**Table S3:** Results of univariate analysis comparing the mean concentrations of metabolites in the CSF obtained from LRRK2 control and LRRK2 PD patients.
P15	**Table S4:** The results of the metabolite set enrichment analysis showing the metabolic pathways that were significantly perturbed when sPD CSF was compared with their respective controls.
P16-20	**Table S5:** Results of univariate analysis comparing the mean concentrations of metabolites in CSF obtained from sPD and LRRK2 PD patients.
P21	**Table S6:** The results of the metabolite set enrichment analysis showing the metabolic pathways that were significantly perturbed when LRKK2 PD CSF was compared with sPD.
P22-26	**Table S7:** Results of univariate analysis comparing the mean concentrations of metabolites in CSF obtained from sPD controls and LRRK2 PD controls.
P27	**Table S8:** Pairwise O-PLS-DA model performance metrics.
P28-29	**Table S9:** Optimized model parameters for each machine learning algorithm evaluated for prediction of sPD and LRRK2 PD in this study.
P30	**Table S10:** The panel of metabolites as chosen using the RFE feature selection algorithm to generate our predictive models
P31-32	**Figure S1:** Representative 1D ^{1}H NMR spectrum of CSF; identified and quantified metabolites are displayed.

P33	**Figure S2:** Investigation of outliers for each group by PCA (a–d) and Q2 vs. T^2 Hotelling plots (e,f).
P34	**Figure S3:** QQ-plots used to determine whether the metabolomics data for each metabolite are normally distributed and/or the corresponding p-value is inflated.
P35-42	**Figure S4:** Box-plots showing pair-wise comparisons of the concentration values of all metabolites in CSF.
P43	**Figure S5:** AUROC plot for the top three ML predictive models for the classification of LRRK2 PD as compared to their corresponding controls.
P44	**Figure S6:** AUROC plot for the top three ML-based predictive models for the classification of sPD as compared to their corresponding controls.
P45	**Figure S7:** AUROC plot for the top three ML-based predictive models for classification of sPD as compared to LRRK2 PD.

Table S1: The mean concentrations of all identified and quantified metabolites for LRRK2 controls and LRRK2 PD, and their corresponding p-values and q-values. The metabolites highlighted in bold are those at significantly different concentrations.

*Metabolites which were non-normally distributed p-values were calculated using the Shapiro–Wilk test.

	**LRRK2 Control**		**LRRK2 PD**		**Statistical Analysis**		
**Label**	**Mean**	**Std**	**Mean**	**Std**	**t-testPvalue**	**Qvalue**	**ShapiroPvalue**
C0	7.5640	0.7915	6.6645	2.3675	0.1154	0.4803	0.0002
C10	0.1457	0.0271	0.1381	0.0270	0.3793	0.7493	0.4724
C10:2	0.0362	0.0095	0.0362	0.0100	1.0000	1.0000	0.8958
C14:2-OH	0.0052	0.0033	0.0054	0.0038	0.8941	0.9930	0.0030
**C16-OH**	**0.0062**	**0.0025**	**0.0040**	**0.0029**	**0.0137**	**0.0223**	**0.0389**
C2	1.7230	0.2609	1.6725	0.4125	0.6462	0.9047	0.4994
C3	0.1157	0.0225	0.1055	0.0296	0.2300	0.6764	0.5328
C4	0.0971	0.0302	0.0955	0.0317	0.8709	0.9930	0.2585
C4:1	0.0239	0.0097	0.0201	0.0140	0.3232	0.7175	0.0192
C5	0.0853	0.1145	0.0576	0.0304	0.3025	0.6957	0.0000
C5-DC (C6-OH)	0.0246	0.0127	0.0203	0.0116	0.2699	0.6835	0.0551
C5-OH (C3-DC-M)	0.0676	0.0282	0.0575	0.0269	0.2558	0.6835	0.4762
C7-DC	0.0076	0.0049	0.0058	0.0050	0.2566	0.6835	0.0098
C9	0.0275	0.0079	0.0216	0.0108	0.0575	0.3957	0.4546
Dimethyl sulfone.1	9.1147	4.9093	15.4085	19.3427	0.1665	0.5712	0.0000
Ethanol	61.9150	79.3710	23.3150	11.0145	0.0376	0.3188	0.0000
Isopropyl alcohol	7.5820	12.3400	2.4065	0.8490	0.0690	0.4116	0.0000
Methanol	432.8303	372.7052	716.3382	959.2531	0.2255	0.6764	0.0000
Propylene glycol	0.1728	0.2077	0.2827	0.1785	0.0805	0.4322	0.0000
Trp	3.7758	0.9294	3.8547	1.3585	0.8314	0.9651	0.3229
Myo-inositol	85.3616	19.6186	85.5548	10.1697	0.9690	1.0000	0.0180
Creatine	31.9261	5.7921	33.1441	5.5023	0.4995	0.8199	0.3545
Creatinine	71.9600	12.1944	70.5550	12.8231	0.7245	0.9379	0.3644
**Dopamine**	**0.0076**	**0.0055**	**0.0131**	**0.0089**	**0.0244**	**0.2866**	**0.0018**
Histamine	5.4497	5.0405	5.5947	5.1046	0.9285	0.9930	0.0001
**Putrescine**	**0.1319**	**0.0337**	**0.2047**	**0.0685**	**0.0001**	**0.0206**	**0.0145**

Sarcosine	1.4526	0.4512	1.7607	0.8325	0.1539	0.5506	0.0001
SDMA	0.1489	0.0409	0.1569	0.0423	0.5465	0.8199	0.3414
t4-OH-Pro	0.6803	0.2166	0.6662	0.1598	0.8161	0.9651	0.2762
Taurine	9.2390	1.9523	8.7800	1.9925	0.4663	0.8199	0.0867
Pyroglutamic acid	60.0908	13.7298	69.7420	13.0648	0.0285	0.2866	0.5625
Urea	152.3632	71.7065	120.8357	61.0592	0.1426	0.5341	0.0003
Ala	77.5850	201.0863	37.6600	14.9355	0.3815	0.7493	0.0000
Arg	22.7700	3.7910	25.6650	6.4101	0.0902	0.4402	0.9087
**Asn**	**7.0120**	**1.2122**	**8.3735**	**1.2159**	**0.0011**	**0.0850**	**0.6623**
Asp	10.5517	16.1031	7.1347	6.9215	0.4030	0.7544	0.0000
Gln	822.1000	246.6783	878.7000	424.5502	0.6092	0.8679	0.0001
Gly	7.9330	2.0113	8.7725	2.6470	0.2658	0.6835	0.0176
His	15.6635	2.2975	18.3783	4.9689	0.0326	0.3090	0.0002
Leu	14.9190	4.4194	15.5565	5.7362	0.6960	0.9264	0.0001
**L-Glutamine**	**43.1854**	**9.7439**	**54.6631**	**16.9382**	**0.0124**	**0.0623**	**0.0363**
Lys	38.2750	8.8781	38.3050	8.0157	0.9911	1.0000	0.4616
**Met**	**3.7040**	**0.7461**	**4.4695**	**1.1211**	**0.0152**	**0.0223**	**0.0094**
Orn	4.9225	1.3128	6.3245	1.9638	0.0115	0.2227	0.0113
Ser	30.9850	6.4278	34.0950	6.4972	0.1363	0.5227	0.6391
**Thr**	**33.5350**	**5.9818**	**39.6450**	**7.2261**	**0.0060**	**0.0323**	**0.6003**
Trp	2.2756	0.6153	2.4530	0.5168	0.3298	0.7175	0.0017
Tyr	9.4930	2.5680	12.3845	4.6551	0.0198	0.2660	0.0000
Val	18.4700	5.2157	19.1400	6.7664	0.7277	0.9379	0.0171
Ile	6.2197	3.4536	5.8129	3.7693	0.7239	0.9379	0.0000
CA	0.0065	0.0074	0.0115	0.0122	0.1250	0.4908	0.0000
CDCA	0.0065	0.0109	0.0130	0.0141	0.1080	0.4803	0.0000
DCA	0.0143	0.0136	0.0319	0.0317	0.0276	0.2866	0.0000
GCA	0.0001	0.0002	0.0035	0.0129	0.2476	0.6835	0.0000
GCDCA	0.0094	0.0057	0.0163	0.0180	0.1110	0.4803	0.0000
**GDCA**	**0.0018**	**0.0018**	**0.0048**	**0.0067**	**0.0590**	**0.3957**	**0.0000**
GUDCA	0.0017	0.0010	0.0019	0.0016	0.5532	0.8199	0.0000
HDCA	0.0045	0.0060	0.0035	0.0036	0.5291	0.8199	0.0000
LCA	0.0012	0.0035	0.0010	0.0025	0.7959	0.9563	0.0000

MCA(b)	0.0001	0.0002	0.0004	0.0007	0.0351	0.3142	0.0000
MCA(o)	0.0012	0.0018	0.0011	0.0018	0.9314	0.9930	0.0000
**TCA**	**0.0094**	**0.0002**	**0.0009**	**0.0019**	**0.0493**	**0.3778**	**0.0000**
TCDCA	0.0017	0.0011	0.0022	0.0013	0.2432	0.6835	0.0122
TDCA	0.0049	0.0030	0.0055	0.0029	0.5207	0.8199	0.0016
TMCA(a+b)	0.0134	0.0005	0.0134	0.0006	0.7739	0.9563	0.0000
TUDCA	0.0041	0.0002	0.0041	0.0003	0.5602	0.8199	0.0000
UDCA	0.0071	0.0058	0.0087	0.0061	0.3846	0.7493	0.0191
Dimethylamine	1.8605	0.9227	1.8716	1.4451	0.9771	1.0000	0.0000
Acetone	4.6190	2.2216	4.3898	1.9333	0.7297	0.9379	0.3470
Ascorbate	138.1400	21.4151	145.3800	24.7369	0.3286	0.7175	0.0630
cis-Aconitate	2.8900	1.0213	3.1750	1.0672	0.3936	0.7493	0.2485
2-Aminobutyrate	6.9000	4.4482	5.3400	3.6151	0.2311	0.6764	0.0000
2-Hydroxybutyric acid	23.7457	6.9783	20.4286	6.0432	0.1163	0.4803	0.6756
2-Hydroxyisovalerate	3.5283	1.1531	3.9484	2.5522	0.5063	0.8199	0.0000
2-Oxoglutarate	7.7350	2.1261	8.1750	1.6714	0.4713	0.8199	0.4158
**3-Hydroxybutyric acid**	**17.6058**	**11.5855**	**12.6481**	**4.0959**	**0.0791**	**0.0432**	**0.0000**
3-Hydroxyisobutyrate	10.1800	2.1118	9.4800	2.6957	0.3664	0.7493	0.1068
3-Hydroxyisovalerate	2.9500	1.0938	2.8300	1.3967	0.7639	0.9534	0.0042
3-Hydroxyisovaleric acid	2.9645	1.2457	2.9603	1.5409	0.9926	1.0000	0.0018
Acetic acid	31.0011	11.3042	30.1263	4.6454	0.7507	0.9501	0.0000
Acetoacetate	5.5904	1.6029	5.6779	1.4719	0.8583	0.9871	0.4822
Citric acid	149.1080	38.2042	157.3319	30.6221	0.4572	0.8199	0.5977
Formate	31.3043	5.4431	29.9754	7.3111	0.5183	0.8199	0.0002
**L-Lactic acid**	**1346.9577**	**184.8950**	**1537.9513**	**361.6891**	**0.0422**	**0.0945**	**0.0002**
L-Phenylalanine	5.9314	1.5825	5.9951	1.6860	0.9026	0.9930	0.8749
Malonate	8.0450	2.0431	8.6400	2.7708	0.4443	0.8129	0.0184
Pantothenate	10.0850	2.7810	8.8250	2.8711	0.1667	0.5712	0.6203
Pyruvic acid	14.7720	11.0199	17.2167	15.9741	0.5765	0.8362	0.0001
Succinate	10.9358	3.5668	11.5770	3.2635	0.5566	0.8199	0.0175
Acetaminophen	3.2150	2.7141	3.7200	2.7152	0.5598	0.8199	0.0000
Choline	0.8300	0.4843	1.1353	0.6049	0.0861	0.4402	0.0001

lysoPC a C26:1	0.0114	0.0127	0.0106	0.0110	0.8222	0.9651	0.0000
lysoPC a C28:0	0.0684	0.0284	0.0716	0.0197	0.6861	0.9264	0.0128
lysoPC a C28:1	0.0250	0.0205	0.0249	0.0209	0.9879	1.0000	0.0001
PC aa C28:1	0.0314	0.0100	0.0352	0.0134	0.3144	0.7130	0.1490
PC aa C30:0	0.1447	0.0346	0.1493	0.0315	0.6661	0.9168	0.3999
PC aa C32:0	0.8991	0.2669	0.9194	0.2079	0.7899	0.9563	0.1535
PC aa C32:1	0.3593	0.0973	0.3625	0.0942	0.9164	0.9930	0.1076
PC aa C32:2	0.0197	0.0194	0.0161	0.0112	0.4774	0.8199	0.0024
PC aa C34:1	5.0525	1.5010	5.2595	1.5108	0.6663	0.9168	0.2255
**PC aa C34:2**	**1.1150**	**0.1039**	**1.0176**	**0.1563**	**0.0258**	**0.0866**	**0.0344**
PC aa C34:4	0.0142	0.0040	0.0128	0.0067	0.4083	0.7557	0.1116
PC aa C36:0	0.1747	0.0312	0.1760	0.0326	0.8982	0.9930	0.6430
PC aa C36:1	0.6716	0.1784	0.7109	0.1911	0.5055	0.8199	0.4740
PC aa C36:2	0.5483	0.1482	0.6017	0.2339	0.3934	0.7493	0.0001
PC aa C36:4	0.4630	0.1880	0.4570	0.1830	0.9197	0.9930	0.0002
PC aa C36:5	0.0339	0.0079	0.0309	0.0096	0.2798	0.6858	0.0003
PC aa C36:6	0.0032	0.0021	0.0030	0.0025	0.7859	0.9563	0.0062
PC aa C38:3	0.2231	0.0899	0.2264	0.0918	0.9092	0.9930	0.1442
PC aa C38:4	0.5471	0.1859	0.5464	0.1666	0.9901	1.0000	0.3811
PC aa C38:5	0.1303	0.0523	0.1251	0.0525	0.7554	0.9501	0.0003
PC aa C38:6	0.1865	0.0731	0.1975	0.0908	0.6755	0.9216	0.0016
PC aa C40:3	0.0128	0.0065	0.0108	0.0068	0.3463	0.7434	0.0149
PC aa C40:4	0.0490	0.0224	0.0535	0.0170	0.4840	0.8199	0.3634
PC aa C40:5	0.0408	0.0175	0.0372	0.0175	0.5255	0.8199	0.4347
PC aa C40:6	0.2308	0.0432	0.2528	0.0595	0.1889	0.6165	0.0030
PC aa C42:2	0.0211	0.0071	0.0205	0.0059	0.7918	0.9563	0.0308
PC ae C30:1	0.0060	0.0033	0.0072	0.0049	0.3878	0.7493	0.1291
PC ae C32:1	0.0577	0.0241	0.0593	0.0204	0.8167	0.9651	0.1742
PC ae C32:2	0.0158	0.0078	0.0138	0.0067	0.3918	0.7493	0.1004
PC ae C34:0	0.0300	0.0128	0.0297	0.0102	0.9243	0.9930	0.1300
PC ae C34:1	0.2016	0.0633	0.2084	0.0614	0.7340	0.9379	0.0324
PC ae C34:2	0.1569	0.0571	0.1580	0.0586	0.9502	0.9999	0.0905
PC ae C34:3	0.0186	0.0084	0.0200	0.0091	0.6165	0.8707	0.1194

**PC ae C36:1**	**0.4928**	**0.0479**	**0.4564**	**0.0679**	**0.0577**	**0.0957**	**0.8053**
**PC ae C36:2**	**0.1233**	**0.0115**	**0.1105**	**0.0171**	**0.0081**	**0.0227**	**0.0465**
PC ae C36:3	0.0323	0.0123	0.0314	0.0145	0.8332	0.9651	0.1397
PC ae C36:4	0.0585	0.0215	0.0535	0.0259	0.5065	0.8199	0.7408
PC ae C36:5	0.0503	0.0201	0.0504	0.0207	0.9939	1.0000	0.0127
PC ae C38:0	0.0189	0.0061	0.0159	0.0036	0.0667	0.4116	0.0008
**PC ae C38:1**	**0.1588**	**0.0207**	**0.1359**	**0.0292**	**0.0068**	**0.0223**	**0.3025**
PC ae C38:2	0.1003	0.0224	0.0870	0.0216	0.0636	0.4098	0.1915
**PC ae C38:3**	**0.0986**	**0.0117**	**0.0824**	**0.0256**	**0.0143**	**0.0223**	**0.0001**
PC ae C38:4	0.0459	0.0201	0.0522	0.0241	0.3756	0.7493	0.0028
PC ae C38:5	0.0658	0.0217	0.0550	0.0210	0.1199	0.4826	0.0661
PC ae C38:6	0.0188	0.0115	0.0228	0.0123	0.2939	0.6858	0.7389
PC ae C40:1	0.0205	0.0085	0.0203	0.0083	0.9253	0.9930	0.0366
PC ae C40:2	0.0400	0.0062	0.0348	0.0064	0.0129	0.2227	0.7551
**PC ae C40:3**	**0.0460**	**0.0149**	**0.0409**	**0.0134**	**0.0267**	**0.0684**	**0.0015**
PC ae C40:4	0.0315	0.0134	0.0294	0.0081	0.5435	0.8199	0.5508
PC ae C40:6	0.0220	0.0083	0.0218	0.0096	0.9442	0.9999	0.5672
PC ae C44:6	0.0117	0.0048	0.0129	0.0040	0.3956	0.7493	0.0004
H1	11859.2500	2441.6785	13003.5000	2978.6285	0.1919	0.6165	0.1441
**Xanthine**	**0.8590**	**0.2287**	**0.7312**	**0.2114**	**0.0742**	**0.4269**	**0.3569**
SM (OH) C14:1	0.0388	0.0115	0.0449	0.0168	0.1880	0.6165	0.2069
SM (OH) C16:1	0.0370	0.0115	0.0400	0.0182	0.5304	0.8199	0.0625
SM (OH) C22:1	0.0352	0.0083	0.0421	0.0161	0.0958	0.4434	0.0012
SM (OH) C22:2	0.0433	0.0201	0.0574	0.0312	0.0964	0.4434	0.0001
SM C16:0	0.8067	0.2742	0.9141	0.3458	0.2833	0.6858	0.5641
SM C16:1	0.0888	0.0293	0.1068	0.0358	0.0900	0.4402	0.1691
SM C18:0	0.8245	0.2461	0.9453	0.3274	0.1953	0.6165	0.3750
SM C18:1	0.2086	0.0667	0.2346	0.0755	0.2558	0.6835	0.4190
SM C20:2	0.0013	0.0023	0.0025	0.0035	0.2078	0.6433	0.0000
SM C24:0	0.1235	0.0483	0.1339	0.0588	0.5429	0.8199	0.6208
SM C24:1	0.4527	0.1216	0.5088	0.2016	0.2933	0.6858	0.0300
SM C26:0	0.0055	0.0034	0.0051	0.0030	0.6962	0.9264	0.1606
SM C26:1	0.0058	0.0055	0.0079	0.0061	0.2717	0.6835	0.0051

Fructose	55.3031	19.0176	61.4039	16.6400	0.2871	0.6858	0.1326
Mannose	23.1880	4.7289	24.3184	8.3913	0.6028	0.8665	0.0001
Glycerol	186.7088	59.4581	175.2335	57.0755	0.5372	0.8199	0.5866
D-Glucose	2165.1198	380.7512	2333.5176	349.5871	0.1533	0.5506	0.0077

Table S2: Results of the metabolite set enrichment analysis showing which metabolic pathways that were significantly perturbed in LRKK2 PD CSF when compared to their corresponding controls.

Pathways	Total	Hits	p values	FDR
Spermidine and spermine biosynthesis	18	3	0.00615	0.04864
Fatty Acid metabolism	43	3	0.00287	0.04562
Mitochondrial Beta oxidation of LCFA[a]	28	1	0.02934	0.03437
Mitochondrial Beta oxidation of SCFA[b]	27	1	0.03869	0.01215
Methionine metabolism	43	6	0.03218	0.02929
Bile Acid Biosynthesis	65	2	0.04376	0.02988
Inositol metabolism	74	4	0.04769	0.2873
Beta oxidation of very LCFA[a]	17	2	0.04895	0.57938
Purine metabolism	11	1	0.04775	0.37697
Urea cycle	19	6	0.05831	0.67286
Taurine and Hypotaurine Metabolism	12	1	0.06100	0.70102

[a] Long chain fatty acid.

[b] Short chain fatty acid.

Table S3: The mean concentrations of all identified and quantified metabolites for sPD controls, sPD and their corresponding p-values and q-values. The metabolites highlighted in bold are those at significantly different concentrations.

*Metabolites which were non-normally distributed p-values were calculated using the Shapiro–Wilk test.

	sPD Control		sPD		Statistical Analysis		
**Label**	**Mean**	**Std**	**Mean**	**Std**	**t-testPvalue**	**Qvalue**	**ShapiroPvalue**
**C0**	**7.516**	**1.537**	**7.074**	**2.9413**	**0.0555**	**0.0968**	**0.0003**
C10	0.152	0.027	0.142	0.0207	0.2076	0.9270	0.0419
C10:2	0.035	0.007	0.037	0.0097	0.5781	0.9677	0.4235
**C14:2-OH**	**0.006**	**0.003**	**0.004**	**0.0036**	**0.0469**	**0.0927**	**0.0018**
**C16-OH**	**0.003**	**0.004**	**0.003**	**0.0028**	**0.0378**	**0.0968**	**0.0000**
C2	1.511	0.262	1.689	0.3387	0.0715	0.9270	0.7369
C3	0.114	0.026	0.112	0.0383	0.8742	0.9719	0.0402
C4	0.099	0.023	0.102	0.0249	0.6642	0.9677	0.0000
C4:1	0.019	0.012	0.021	0.0108	0.6719	0.9677	0.2997
C5	0.070	0.019	0.075	0.0209	0.4851	0.9677	0.0157
C5-DC (C6-OH)	0.022	0.011	0.023	0.0126	0.8542	0.9719	0.1622
C5-OH (C3-DC-M)	0.062	0.019	0.061	0.0243	0.8802	0.9719	0.5825
C7-DC	0.009	0.005	0.007	0.0050	0.2082	0.9270	0.0962
C9	0.022	0.008	0.024	0.0108	0.4477	0.9610	0.3044
Dimethyl sulfone.1	9.335	5.974	12.391	6.2499	0.1223	0.9270	0.0003
Ethanol	18.280	9.966	73.285	191.3642	0.2070	0.9270	0.0000
Isopropyl alcohol	1.770	0.753	84.259	361.9489	0.3145	0.9270	0.0000
Methanol	225.355	274.341	378.311	351.8160	0.1335	0.9270	0.0000
Propylene glycol	0.435	0.760	0.802	2.0735	0.4622	0.9645	0.0000
Trp	5.260	6.195	3.597	1.1350	0.2449	0.9270	0.0000
**Myo-inositol**	**87.670**	**17.284**	**94.481**	**19.0134**	**0.0243**	**0.0927**	**0.0197**

Creatine	31.525	4.180	33.146	5.3577	0.2929	0.9270	0.0979
Creatinine	68.340	9.821	67.780	12.5600	0.8760	0.9719	0.4173
Dopamine	0.010	0.005	0.012	0.0061	0.2431	0.9270	0.0000
Histamine	3.270	2.648	5.710	6.7862	0.1424	0.9270	0.0000
**Putrescine**	**0.133**	**0.048**	**0.162**	**0.0546**	**0.0777**	**0.0927**	**0.0070**
Sarcosine	1.620	0.518	1.665	0.7586	0.8283	0.9719	0.0002
SDMA	0.156	0.039	0.158	0.0306	0.8722	0.9719	0.0111
t4-OH-Pro	0.672	0.311	0.646	0.1985	0.7589	0.9677	0.0028
Taurine	8.497	1.785	7.784	2.2162	0.2692	0.9270	0.0795
Pyroglutamic acid	67.730	17.332	69.668	15.3749	0.7104	0.9677	0.1274
Urea	305.365	614.697	135.707	69.3058	0.2275	0.9270	0.0000
Ala	35.520	9.389	36.885	15.2885	0.7355	0.9677	0.0002
Arg	24.070	4.088	23.610	5.1290	0.7555	0.9677	0.0298
Asn	7.359	1.315	7.833	1.7213	0.3340	0.9270	0.0630
Asp	13.047	11.114	28.304	82.0962	0.4274	0.9479	0.0000
Gln	815.450	152.453	737.250	341.3445	0.3554	0.9277	0.0000
Gly	7.555	1.985	12.630	22.3671	0.3185	0.9270	0.0000
**His**	**13.427**	**6.035**	**17.214**	**7.1285**	**0.0777**	**0.0927**	**0.0000**
Leu	16.835	6.608	17.348	6.6228	0.8078	0.9719	0.0000
L-Glutamine	50.365	17.184	47.368	13.9630	0.5485	0.9677	0.1248
Lys	39.645	9.767	39.490	7.9216	0.9563	0.9719	0.0333
**Met**	**3.543**	**0.614**	**4.079**	**1.1650**	**0.0766**	**0.0893**	**0.0001**
Orn	5.255	1.354	7.654	10.4987	0.3173	0.9270	0.0000
Ser	28.935	5.121	27.200	8.4121	0.4357	0.9479	0.0008
Thr	32.600	7.983	37.315	10.9019	0.1269	0.9270	0.1939
Trp	2.241	0.450	2.544	0.9251	0.1949	0.9270	0.0000
Tyr	9.312	2.027	10.013	3.2771	0.4209	0.9479	0.0448
Val	19.740	7.052	20.415	6.9983	0.7629	0.9677	0.0010
Ile	7.900	3.716	6.452	3.3387	0.2028	0.9270	0.0022
CA	0.010	0.012	0.013	0.0124	0.3573	0.9277	0.0000
CDCA	0.010	0.012	0.012	0.0125	0.5696	0.9677	0.0000
DCA	0.022	0.015	0.020	0.0199	0.7741	0.9719	0.0040
GCA	0.002	0.004	0.001	0.0017	0.3231	0.9270	0.0000

**GCDCA**	**0.020**	**0.018**	**0.010**	**0.0055**	**0.0235**	**0.0704**	**0.0000**
GDCA	0.004	0.005	0.002	0.0026	0.3438	0.9277	0.0000
GUDCA	0.002	0.001	0.002	0.0009	0.6323	0.9677	0.0000
HDCA	0.004	0.005	0.004	0.0051	0.8824	0.9719	0.0000
LCA	0.001	0.003	0.000	0.0009	0.1037	0.9270	0.0000
MCA(b)	0.000	0.000	0.000	0.0007	0.3645	0.9293	0.0000
MCA(o)	0.001	0.001	0.002	0.0029	0.2172	0.9270	0.0000
TCA	0.000	0.001	0.000	0.0000	0.1785	0.9270	0.0000
TCDCA	0.002	0.001	0.003	0.0013	0.2933	0.9270	0.0120
TDCA	0.005	0.002	0.004	0.0032	0.7411	0.9677	0.0018
TMCA(a+b)	0.013	0.001	0.013	0.0005	0.5602	0.9677	0.0000
TUDCA	0.004	0.000	0.004	0.0002	0.5602	0.9677	0.0000
UDCA	0.006	0.005	0.006	0.0049	0.9255	0.9719	0.0021
Dimethylamine	1.715	0.671	1.857	0.5870	0.4804	0.9677	0.0005
**Acetone**	**3.625**	**2.520**	**5.023**	**2.2748**	**0.0341**	**0.0704**	**0.0006**
Ascorbate	142.965	30.960	140.855	15.3580	0.7863	0.9719	0.0019
cis-Aconitate	2.990	0.896	2.740	0.9366	0.3937	0.9293	0.0771
2-Aminobutyrate	6.700	6.430	6.475	4.0182	0.8951	0.9719	0.0000
2-Hydroxybutyric acid	22.730	7.127	25.831	10.8912	0.2934	0.9270	0.0001
**2-Hydroxyisovalerate**	**3.845**	**1.289**	**4.648**	**2.9030**	**0.0265**	**0.0927**	**0.0000**
2-Oxoglutarate	8.525	2.257	8.610	1.8284	0.8966	0.9719	0.1623
**3-Hydroxybutyric acid**	**9.315**	**3.745**	**17.179**	**10.5270**	**0.0032**	**0.0477**	**0.0000**
3-Hydroxyisobutyrate	11.140	2.568	12.105	6.6828	0.5502	0.9677	0.0000
3-Hydroxyisovalerate	3.600	1.421	3.140	1.4691	0.3205	0.9270	0.0001
3-Hydroxyisovaleric acid	3.675	1.226	3.090	1.5801	0.1988	0.9270	0.0203
Acetic acid	30.405	6.394	34.561	16.6800	0.3047	0.9270	0.0000
Acetoacetate	5.100	1.599	6.405	3.7859	0.1639	0.9270	0.0000
**Citric acid**	**147.975**	**30.763**	**168.096**	**40.4322**	**0.0446**	**0.0927**	**0.0683**
Formate	30.155	4.720	30.107	6.9181	0.9795	0.9795	0.5875
**L-Lactic acid**	**1381.950**	**194.326**	**1511.529**	**241.0402**	**0.0397**	**0.0927**	**0.0274**
L-Phenylalanine	6.655	5.401	6.106	1.8693	0.6698	0.9677	0.0000
Malonate	8.285	1.993	7.770	2.3775	0.4624	0.9645	0.1187
Pantothenate	9.640	3.195	10.375	4.0676	0.5289	0.9677	0.0011

Pyruvic acid	14.910	9.001	13.151	11.8267	0.5996	0.9677	0.0002
Succinate	11.515	3.008	11.629	2.9807	0.9048	0.9719	0.3544
Acetaminophen	4.075	4.376	2.780	0.9088	0.2029	0.9270	0.0000
Choline	1.030	0.782	0.876	0.5128	0.4673	0.9645	0.0000
lysoPC a C26:1	0.012	0.012	0.018	0.0128	0.1612	0.9270	0.0040
lysoPC a C28:0	0.065	0.027	0.064	0.0389	0.9404	0.9719	0.0214
lysoPC a C28:1	0.032	0.023	0.031	0.0206	0.9598	0.9719	0.0063
**PC aa C28:1**	**0.027**	**0.011**	**0.033**	**0.0111**	**0.0452**	**0.0927**	**0.4571**
PC aa C30:0	0.134	0.028	0.146	0.0277	0.1740	0.9270	0.8363
PC aa C32:0	0.818	0.213	0.845	0.2323	0.7035	0.9677	0.5175
PC aa C32:1	0.330	0.084	0.337	0.1008	0.8044	0.9719	0.4761
PC aa C32:2	0.018	0.012	0.015	0.0108	0.5170	0.9677	0.1322
PC aa C34:1	4.576	1.234	4.822	1.3745	0.5542	0.9677	0.8106
PC aa C34:2	1.051	0.100	1.030	0.1268	0.5684	0.9677	0.1387
PC aa C34:4	0.014	0.005	0.011	0.0057	0.1671	0.9270	0.0009
PC aa C36:0	0.179	0.037	0.175	0.0453	0.7069	0.9677	0.0730
PC aa C36:1	0.620	0.182	0.640	0.1579	0.7148	0.9677	0.5553
PC aa C36:2	0.556	0.180	0.532	0.1705	0.6701	0.9677	0.0000
PC aa C36:4	0.480	0.170	0.410	0.1553	0.1822	0.9270	0.0688
PC aa C36:5	0.037	0.025	0.029	0.0089	0.1861	0.9270	0.0000
**PC aa C36:6**	**0.004**	**0.002**	**0.002**	**0.0025**	**0.0314**	**0.0704**	**0.0040**
PC aa C38:3	0.223	0.100	0.230	0.1000	0.8308	0.9719	0.2062
PC aa C38:4	0.536	0.149	0.510	0.1584	0.5939	0.9677	0.9092
PC aa C38:5	0.127	0.053	0.121	0.0475	0.7052	0.9677	0.0087
PC aa C38:6	0.202	0.101	0.178	0.0690	0.3965	0.9293	0.0047
**PC aa C40:3**	**0.010**	**0.007**	**0.011**	**0.0049**	**0.0292**	**0.0807**	**0.0058**
PC aa C40:4	0.047	0.018	0.053	0.0191	0.3788	0.9293	0.7605
PC aa C40:5	0.035	0.021	0.032	0.0180	0.6235	0.9677	0.0730
PC aa C40:6	0.240	0.053	0.239	0.0524	0.9313	0.9719	0.6780
PC aa C42:2	0.020	0.008	0.023	0.0071	0.2349	0.9270	0.0201
PC ae C30:1	0.006	0.004	0.006	0.0047	0.9112	0.9719	0.1486
PC ae C32:1	0.054	0.021	0.056	0.0182	0.7506	0.9677	0.4956
PC ae C32:2	0.015	0.008	0.015	0.0090	0.9697	0.9758	0.1011

PC ae C34:0	0.028	0.011	0.027	0.0116	0.7954	0.9719	0.0389
PC ae C34:1	0.188	0.051	0.210	0.0628	0.2487	0.9270	0.1776
PC ae C34:2	0.155	0.065	0.168	0.0681	0.5553	0.9677	0.6378
PC ae C34:3	0.021	0.005	0.020	0.0074	0.9016	0.9719	0.0274
PC ae C36:1	0.466	0.039	0.462	0.0657	0.8232	0.9719	0.2501
PC ae C36:2	0.114	0.014	0.115	0.0159	0.9327	0.9719	0.1051
PC ae C36:3	0.030	0.013	0.031	0.0109	0.8053	0.9719	0.1187
PC ae C36:4	0.057	0.022	0.056	0.0216	0.9425	0.9719	0.7605
PC ae C36:5	0.046	0.017	0.048	0.0172	0.8135	0.9719	0.3319
PC ae C38:0	0.018	0.005	0.017	0.0053	0.7536	0.9677	0.6015
PC ae C38:1	0.136	0.027	0.132	0.0384	0.7060	0.9677	0.7290
PC ae C38:2	0.092	0.016	0.089	0.0260	0.6908	0.9677	0.0680
PC ae C38:3	0.091	0.008	0.092	0.0123	0.8464	0.9719	0.8713
PC ae C38:4	0.044	0.019	0.049	0.0220	0.4320	0.9479	0.0006
PC ae C38:5	0.060	0.021	0.052	0.0134	0.1317	0.9270	0.7289
PC ae C38:6	0.021	0.012	0.023	0.0101	0.5451	0.9677	0.2839
PC ae C40:1	0.021	0.007	0.018	0.0076	0.2343	0.9270	0.0007
PC ae C40:2	0.036	0.005	0.036	0.0102	0.9538	0.9719	0.0000
PC ae C40:3	0.044	0.013	0.042	0.0178	0.7532	0.9677	0.0001
PC ae C40:4	0.031	0.010	0.035	0.0102	0.2087	0.9270	0.0326
PC ae C40:6	0.019	0.008	0.020	0.0107	0.7634	0.9677	0.3932
PC ae C44:6	0.009	0.007	0.011	0.0066	0.3983	0.9293	0.0463
H1	11221.789	1777.259	10986.200	1744.8288	0.6786	0.9677	0.3275
Xanthine	1.190	1.487	0.887	0.3147	0.3789	0.9293	0.0000
SM (OH) C14:1	0.042	0.014	0.039	0.0145	0.5501	0.9677	0.0071
SM (OH) C16:1	0.033	0.016	0.040	0.0180	0.1877	0.9270	0.0010
SM (OH) C22:1	0.039	0.018	0.044	0.0252	0.5310	0.9677	0.0000
SM (OH) C22:2	0.044	0.020	0.047	0.0302	0.7069	0.9677	0.0001
SM C16:0	0.836	0.251	0.751	0.3113	0.3468	0.9277	0.3325
SM C16:1	0.098	0.035	0.089	0.0306	0.4084	0.9393	0.0311
SM C18:0	0.781	0.279	0.829	0.2542	0.5727	0.9677	0.2314
SM C18:1	0.208	0.076	0.212	0.0579	0.8541	0.9719	0.1199
**SM C20:2**	**0.001**	**0.002**	**0.004**	**0.0055**	**0.0454**	**0.6044**	**0.0000**

SM C24:0	0.145	0.057	0.139	0.0447	0.7266	0.9677	0.8723
SM C24:1	0.464	0.164	0.486	0.1901	0.7006	0.9677	0.5975
**SM C26:0**	**0.005**	**0.005**	**0.004**	**0.0030**	**0.0297**	**0.0704**	**0.0014**
SM C26:1	0.008	0.005	0.007	0.0037	0.3176	0.9270	0.0133
Fructose	58.160	16.345	56.836	10.2266	0.7605	0.9677	0.0012
Mannose	27.155	19.451	22.600	7.2744	0.3328	0.9270	0.0000
Glycerol	153.965	40.720	154.871	46.8308	0.9483	0.9719	0.1398
D-Glucose	2299.345	291.700	2231.196	190.3433	0.3871	0.9293	0.0482

Table S4: Results of the metabolite set enrichment analysis showing which metabolic pathways that were significantly perturbed in sPD CSF when compared to their corresponding controls.

**Pathways**	**Total**	**Hits**	**p values**	**FDR**
Fatty Acid Biosynthesis	35	4	0.00184	0.0757
Ethanol Degradation	19	2	0.00574	0.0437
Ketone Body Metabolism	13	3	0.02987	0.0199
Bile Acid Biosynthesis	65	2	0.03259	0.0122
Propionate Metabolism	40	1	0.04492	0.0293
Taurine and Hypotaurine Metabolism	19	2	0.04598	0.2989
Inositol metabolism	12	7	0.67286	0.2874
Mitochondrial Electron Transport Chain	60	1	0.07424	0.5794
Carnitine Synthesis	19	6	0.08783	0.0721
D-Arginine and D-Ornithine Metabolism	22	6	0.08436	0.7010
Methionine Metabolism	11	6	0.08153	0.8209

Table S5: The mean concentrations of all identified and quantified metabolites for sPD and LRRK2 PD and their corresponding p-values and q-values. The metabolites highlighted in bold are those at significantly different concentrations.

*Metabolites which were non-normally distributed p-values were calculated using the Shapiro–Wilk test.

	**sPD**		**LRRK2 PD**		**Statistical Analysis**		
**Label**	**Mean**	**Std**	**Mean**	**Std**	**t-TestPvalue**	**Qvalue**	**ShapiroPvalue**
C0	7.0735	2.9413	6.6645	2.3675	0.6309	0.8744	0.0026
C10	0.1424	0.0207	0.1381	0.0270	0.5750	0.8664	0.8078
C10:2	0.0367	0.0097	0.0362	0.0100	0.8732	0.9704	0.3111
C14:2-OH	0.0042	0.0036	0.0054	0.0038	0.3270	0.8664	0.0007
C16-OH	0.0028	0.0028	0.0040	0.0029	0.2093	0.8664	0.0002
C2	1.6885	0.3387	1.6725	0.4125	0.8941	0.9779	0.3344
C3	0.1120	0.0383	0.1055	0.0296	0.5519	0.8664	0.1251
C4	0.1024	0.0249	0.0955	0.0317	0.4449	0.8664	0.0155
C4:1	0.0207	0.0108	0.0201	0.0140	0.8800	0.9704	0.0102
**C5**	**0.0749**	**0.0209**	**0.0576**	**0.0304**	**0.0429**	**0.8664**	**0.2108**
C5-DC (C6-OH)	0.0227	0.0126	0.0203	0.0116	0.5433	0.8664	0.0576
C5-OH (C3-DC-M)	0.0611	0.0243	0.0575	0.0269	0.6638	0.8906	0.6819
C7-DC	0.0069	0.0050	0.0058	0.0050	0.5110	0.8664	0.0090
C9	0.0242	0.0108	0.0216	0.0108	0.4600	0.8664	0.2138
Dimethyl sulfone.1	12.3906	6.2499	15.4085	19.3427	0.5107	0.8664	0.0000
Ethanol	73.2850	191.3642	23.3150	11.0145	0.2509	0.8664	0.0000
Isopropyl alcohol	84.2593	361.9489	2.4065	0.8490	0.3182	0.8664	0.0000
Methanol	378.3109	351.8160	716.3382	959.2531	0.1472	0.8664	0.0000
Propylene glycol	0.8018	2.0735	0.2827	0.1785	0.2717	0.8664	0.0000
Trp	3.5967	1.1350	3.8547	1.3585	0.5185	0.8664	0.5216
**Myo-inositol**	**94.4813**	**19.0134**	**85.5548**	**10.1697**	**0.0719**	**0.8664**	**0.0042**
Creatine	33.1458	5.3577	33.1441	5.5023	0.9992	1.0000	0.2188
Creatinine	67.7800	12.5600	70.5550	12.8231	0.4935	0.8664	0.2697
Dopamine	0.0118	0.0061	0.0131	0.0089	0.5785	0.8664	0.0013

Histamine	5.7097	6.7862	5.5947	5.1046	0.9520	0.9907	0.0000
**Putrescine**	**0.1623**	**0.0546**	**0.2047**	**0.0685**	**0.0368**	**0.8664**	**0.0557**
Sarcosine	1.6652	0.7586	1.7607	0.8325	0.7068	0.9288	0.0001
SDMA	0.1581	0.0306	0.1569	0.0423	0.9186	0.9794	0.0608
t4-OH-Pro	0.6462	0.1985	0.6662	0.1598	0.7269	0.9288	0.3661
Taurine	7.7835	2.2162	8.7800	1.9925	0.1431	0.8664	0.0096
Pyroglutamic acid	69.6680	15.3749	69.7420	13.0648	0.9870	0.9994	0.8993
Urea	135.7073	69.3058	120.8357	61.0592	0.4759	0.8664	0.0000
Ala	36.8850	15.2885	37.6600	14.9355	0.8720	0.9704	0.0002
Arg	23.6100	5.1290	25.6650	6.4101	0.2700	0.8664	0.7420
Asn	7.8325	1.7213	8.3735	1.2159	0.2581	0.8664	0.0608
Asp	28.3042	82.0962	7.1347	6.9215	0.2701	0.8664	0.0000
Gln	737.2500	341.3445	878.7000	424.5502	0.2528	0.8664	0.0000
Gly	12.6300	22.3671	8.7725	2.6470	0.4484	0.8664	0.0000
His	17.2143	7.1285	18.3783	4.9689	0.5527	0.8664	0.0000
Leu	17.3475	6.6228	15.5565	5.7362	0.3664	0.8664	0.0002
L-Glutamine	47.3677	13.9630	54.6631	16.9382	0.1455	0.8664	0.3612
Lys	39.4900	7.9216	38.3050	8.0157	0.6409	0.8744	0.5335
Met	4.0790	1.1650	4.4695	1.1211	0.2869	0.8664	0.0008
Orn	7.6535	10.4987	6.3245	1.9638	0.5812	0.8664	0.0000
**Ser**	**27.2000**	**8.4121**	**34.0950**	**6.4972**	**0.0062**	**0.8664**	**0.0082**
Thr	37.3150	10.9019	39.6450	7.2261	0.4306	0.8664	0.7975
Trp	2.5440	0.9251	2.4530	0.5168	0.7031	0.9288	0.0000
Tyr	10.0125	3.2771	12.3845	4.6551	0.0702	0.8664	0.0003
Val	20.4150	6.9983	19.1400	6.7664	0.5615	0.8664	0.0135
Ile	6.4525	3.3387	5.8129	3.7693	0.5733	0.8664	0.0000
CA	0.0133	0.0124	0.0115	0.0122	0.6366	0.8744	0.0001
CDCA	0.0122	0.0125	0.0130	0.0141	0.8503	0.9704	0.0000
DCA	0.0199	0.0199	0.0319	0.0317	0.1596	0.8664	0.0000
GCA	0.0007	0.0017	0.0035	0.0129	0.3435	0.8664	0.0000
GCDCA	0.0102	0.0055	0.0163	0.0180	0.1558	0.8664	0.0000
GDCA	0.0024	0.0026	0.0048	0.0067	0.1342	0.8664	0.0000
GUDCA	0.0021	0.0009	0.0019	0.0016	0.6277	0.8744	0.0000

HDCA	0.0039	0.0051	0.0035	0.0036	0.7772	0.9329	0.0000
LCA	0.0002	0.0009	0.0010	0.0025	0.2179	0.8664	0.0000
MCA(b)	0.0002	0.0007	0.0004	0.0007	0.3639	0.8664	0.0000
MCA(o)	0.0019	0.0029	0.0011	0.0018	0.3331	0.8664	0.0000
**TCA**	**0.0000**	**0.0000**	**0.0009**	**0.0019**	**0.0493**	**0.8664**	**0.0000**
TCDCA	0.0027	0.0013	0.0022	0.0013	0.2415	0.8664	0.0186
TDCA	0.0045	0.0032	0.0055	0.0029	0.3066	0.8664	0.0043
TMCA(a+b)	0.0133	0.0005	0.0134	0.0006	0.5602	0.8664	0.0000
TUDCA	0.0041	0.0002	0.0041	0.0003	0.5602	0.8664	0.0000
UDCA	0.0057	0.0049	0.0087	0.0061	0.0890	0.8664	0.0198
Dimethylamine	1.8570	0.5870	1.8716	1.4451	0.9669	0.9907	0.0000
Acetone	5.0226	2.2748	4.3898	1.9333	0.3492	0.8664	0.0091
Ascorbate	140.8550	15.3580	145.3800	24.7369	0.4913	0.8664	0.0458
cis-Aconitate	2.7400	0.9366	3.1750	1.0672	0.1787	0.8664	0.0543
2-Aminobutyrate	6.4750	4.0182	5.3400	3.6151	0.3536	0.8664	0.0009
**2-Hydroxybutyric acid**	**25.8306**	**10.8912**	**20.4286**	**6.0432**	**0.0599**	**0.8664**	**0.0000**
2-Hydroxyisovalerate	4.6485	2.9030	3.9484	2.5522	0.4230	0.8664	0.0000
2-Oxoglutarate	8.6100	1.8284	8.1750	1.6714	0.4371	0.8664	0.0015
**3-Hydroxybutyric acid**	**17.1790**	**10.5270**	**12.6481**	**4.0959**	**0.0808**	**0.8664**	**0.0000**
3-Hydroxyisobutyrate	12.1050	6.6828	9.4800	2.6957	0.1116	0.8664	0.0000
3-Hydroxyisovalerate	3.1400	1.4691	2.8300	1.3967	0.4982	0.8664	0.0001
3-Hydroxyisovaleric acid	3.0902	1.5801	2.9603	1.5409	0.7938	0.9329	0.0006
Acetic acid	34.5609	16.6800	30.1263	4.6454	0.2592	0.8664	0.0000
Acetoacetate	6.4045	3.7859	5.6779	1.4719	0.4287	0.8664	0.0000
Citric acid	168.0960	40.4322	157.3319	30.6221	0.3486	0.8664	0.2676
Formate	30.1066	6.9181	29.9754	7.3111	0.9538	0.9907	0.0001
L-Lactic acid	1511.5289	241.0402	1537.9513	361.6891	0.7872	0.9329	0.0009
L-Phenylalanine	6.1058	1.8693	5.9951	1.6860	0.8451	0.9704	0.3915
Malonate	7.7700	2.3775	8.6400	2.7708	0.2933	0.8664	0.0301
Pantothenate	10.3750	4.0676	8.8250	2.8711	0.1719	0.8664	0.0002
Pyruvic acid	13.1507	11.8267	17.2167	15.9741	0.3660	0.8664	0.0000
Succinate	11.6290	2.9807	11.5770	3.2635	0.9584	0.9907	0.2807
Acetaminophen	2.7800	0.9088	3.7200	2.7152	0.1503	0.8664	0.0000

Choline	0.8764	0.5128	1.1353	0.6049	0.1526	0.8664	0.0004
lysoPC a C26:1	0.0180	0.0128	0.0106	0.0110	0.0580	0.8664	0.0025
lysoPC a C28:0	0.0644	0.0389	0.0716	0.0197	0.4649	0.8664	0.0161
lysoPC a C28:1	0.0312	0.0206	0.0249	0.0209	0.3390	0.8664	0.0042
PC aa C28:1	0.0334	0.0111	0.0352	0.0134	0.6369	0.8744	0.0545
PC aa C30:0	0.1464	0.0277	0.1493	0.0315	0.7632	0.9329	0.3664
PC aa C32:0	0.8451	0.2323	0.9194	0.2079	0.2929	0.8664	0.3789
PC aa C32:1	0.3373	0.1008	0.3625	0.0942	0.4190	0.8664	0.4653
PC aa C32:2	0.0152	0.0108	0.0161	0.0112	0.7868	0.9329	0.0415
PC aa C34:1	4.8220	1.3745	5.2595	1.5108	0.3442	0.8664	0.7635
PC aa C34:2	1.0301	0.1268	1.0176	0.1563	0.7836	0.9329	0.3301
PC aa C34:4	0.0112	0.0057	0.0128	0.0067	0.4204	0.8664	0.0073
PC aa C36:0	0.1745	0.0453	0.1760	0.0326	0.9050	0.9779	0.5272
PC aa C36:1	0.6396	0.1579	0.7109	0.1911	0.2060	0.8664	0.7257
PC aa C36:2	0.5325	0.1705	0.6017	0.2339	0.2915	0.8664	0.0001
PC aa C36:4	0.4103	0.1553	0.4570	0.1830	0.3892	0.8664	0.0020
PC aa C36:5	0.0288	0.0089	0.0309	0.0096	0.4896	0.8664	0.0002
PC aa C36:6	0.0023	0.0025	0.0030	0.0025	0.3822	0.8664	0.0003
PC aa C38:3	0.2301	0.1000	0.2264	0.0918	0.9049	0.9779	0.0758
PC aa C38:4	0.5103	0.1584	0.5464	0.1666	0.4868	0.8664	0.8618
PC aa C38:5	0.1207	0.0475	0.1251	0.0525	0.7826	0.9329	0.0016
PC aa C38:6	0.1785	0.0690	0.1975	0.0908	0.4596	0.8664	0.0086
PC aa C40:3	0.0106	0.0049	0.0108	0.0068	0.9153	0.9794	0.0134
PC aa C40:4	0.0525	0.0191	0.0535	0.0170	0.8690	0.9704	0.8632
PC aa C40:5	0.0320	0.0180	0.0372	0.0175	0.3563	0.8664	0.3692
PC aa C40:6	0.2388	0.0524	0.2528	0.0595	0.4345	0.8664	0.2029
PC aa C42:2	0.0233	0.0071	0.0205	0.0059	0.1909	0.8664	0.0875
PC ae C30:1	0.0064	0.0047	0.0072	0.0049	0.5999	0.8744	0.0356
PC ae C32:1	0.0561	0.0182	0.0593	0.0204	0.6042	0.8744	0.6042
PC ae C32:2	0.0154	0.0090	0.0138	0.0067	0.5268	0.8664	0.1032
PC ae C34:0	0.0273	0.0116	0.0297	0.0102	0.4915	0.8664	0.0706
PC ae C34:1	0.2096	0.0628	0.2084	0.0614	0.9516	0.9907	0.0349
PC ae C34:2	0.1675	0.0681	0.1580	0.0586	0.6390	0.8744	0.1581

PC ae C34:3	0.0204	0.0074	0.0200	0.0091	0.8798	0.9704	0.0293
PC ae C36:1	0.4620	0.0657	0.4564	0.0679	0.7924	0.9329	0.7883
PC ae C36:2	0.1146	0.0159	0.1105	0.0171	0.4313	0.8664	0.2061
PC ae C36:3	0.0312	0.0109	0.0314	0.0145	0.9707	0.9907	0.3340
PC ae C36:4	0.0560	0.0216	0.0535	0.0259	0.7376	0.9329	0.4804
PC ae C36:5	0.0475	0.0172	0.0504	0.0207	0.6382	0.8744	0.0503
PC ae C38:0	0.0174	0.0053	0.0159	0.0036	0.3013	0.8664	0.1380
PC ae C38:1	0.1321	0.0384	0.1359	0.0292	0.7231	0.9288	0.5645
PC ae C38:2	0.0895	0.0260	0.0870	0.0216	0.7430	0.9329	0.0813
**PC ae C38:3**	**0.0915**	**0.0123**	**0.0824**	**0.0256**	**0.1604**	**0.8664**	**0.0001**
PC ae C38:4	0.0487	0.0220	0.0522	0.0241	0.6393	0.8744	0.0016
PC ae C38:5	0.0515	0.0134	0.0550	0.0210	0.5336	0.8664	0.4485
PC ae C38:6	0.0228	0.0101	0.0228	0.0123	1.0000	1.0000	0.9848
PC ae C40:1	0.0183	0.0076	0.0203	0.0083	0.4439	0.8664	0.0388
PC ae C40:2	0.0357	0.0102	0.0348	0.0064	0.7267	0.9288	0.0002
PC ae C40:3	0.0421	0.0178	0.0409	0.0134	0.8110	0.9408	0.0008
**PC ae C40:4**	**0.0346**	**0.0102**	**0.0294**	**0.0081**	**0.0829**	**0.8664**	**0.4351**
PC ae C40:6	0.0203	0.0107	0.0218	0.0096	0.6550	0.8861	0.4112
PC ae C44:6	0.0113	0.0066	0.0129	0.0040	0.3602	0.8664	0.0614
H1	10986.2000	1744.8288	13003.5000	2978.6285	0.0128	0.8664	0.0116
**Xanthine**	**0.8873**	**0.3147**	**0.7312**	**0.2114**	**0.0733**	**0.8664**	**0.1950**
SM (OH) C14:1	0.0394	0.0145	0.0449	0.0168	0.2791	0.8664	0.0052
SM (OH) C16:1	0.0398	0.0180	0.0400	0.0182	0.9723	0.9907	0.0113
SM (OH) C22:1	0.0437	0.0252	0.0421	0.0161	0.8122	0.9408	0.0000
SM (OH) C22:2	0.0471	0.0302	0.0574	0.0312	0.2954	0.8664	0.0003
SM C16:0	0.7507	0.3113	0.9141	0.3458	0.1246	0.8664	0.3318
SM C16:1	0.0893	0.0306	0.1068	0.0358	0.1042	0.8664	0.0522
SM C18:0	0.8288	0.2542	0.9453	0.3274	0.2164	0.8664	0.4795
SM C18:1	0.2124	0.0579	0.2346	0.0755	0.3036	0.8664	0.2905
SM C20:2	0.0038	0.0055	0.0025	0.0035	0.3981	0.8664	0.0000
SM C24:0	0.1390	0.0447	0.1339	0.0588	0.7614	0.9329	0.8559
SM C24:1	0.4857	0.1901	0.5088	0.2016	0.7108	0.9288	0.1291
SM C26:0	0.0040	0.0030	0.0051	0.0030	0.2588	0.8664	0.1191

SM C26:1	0.0070	0.0037	0.0079	0.0061	0.5760	0.8664	0.0038
Fructose	56.8362	10.2266	61.4039	16.6400	0.3022	0.8664	0.0230
Mannose	22.5995	7.2744	24.3184	8.3913	0.4930	0.8664	0.0029
Glycerol	154.8710	46.8308	175.2335	57.0755	0.2250	0.8664	0.5937
D-Glucose	2231.1958	190.3433	2333.5176	349.5871	0.2575	0.8664	0.0634

Table 6: Results of the metabolite set enrichment analysis showing which metabolic pathways that were significantly perturbed when LRRK2 PD and sPD CSF where compared.

Pathways	Total	Hits	p values	FDR
Propionate metabolism	42	3	0.00192	0.05437
Inositol metabolism	33	1	0.00367	0.05249
Inositol phosphate metabolism	26	1	0.02359	0.04375
Phosphatidylinositol Phosphate Metabolism	17	1	0.02883	0.12154
Valine, Leucine and Isoleucine Degradation	60	7	0.00308	0.04578
Beta-Alanine Metabolism	34	4	0.01144	0.25625
Tyrosine metabolism	72	6	0.04769	0.18840
Phytanic Acid Peroxisomal Oxidation	26	3	0.01161	0.3128
Lysine degradation	30	2	0.02845	0.8493
Phenylalanine and Tyrosine Metabolism	19	6	0.05129	0.7856

Table S7: The mean concentrations of all identified and quantified metabolites for sPD controls and LRRK2 PD controls and their corresponding p-values and q-values. The metabolites highlighted in bold are those at significantly different concentrations.

*Metabolites which were non-normally distributed p-values were calculated using the Shapiro–Wilk test.

	**sPD Control**		**LRRK2 Control**		**Statistical Analaysis**		
**Label**	**Mean**	**Std**	**Mean**	**Std**	**t-testPvalue**	**Qvalue**	**ShapiroPvalue**
C0	7.516	1.5372	7.564	0.792	0.9008	0.9661	0. 5
C10	0.152	0.0268	0.146	0.027	0.4567	0.9159	0. 232
C10:2	0.035	0.0069	0.036	0.009	0.7060	0.9509	0.9064
C14:2-OH	0.006	0.0034	0.005	0.003	0.4044	0.8799	0.0101
C16-OH	0.003	0.0039	0.006	0.003	0.80	0.4313	0. 41
C2	1.511	0.2619	1.723	0.261	0.0144	0.4894	0.6029
C3	0.114	0.0260	0.116	0.022	0.7961	0.9657	0.6224
C4	0.099	0.0228	0.097	0.030	0.8097	0.9657	0.2
C4:1	0.019	0.0121	0.024	0.010	0.1786	0.8709	0.3038
C5	0.070	0.0194	0.085	0.115	0.5696	0.9356	0.1000
C5-DC (C6-OH)	0.022	0.0113	0.025	0.013	0.4891	0.9263	0.1289
C5-OH (C3-DC-M)	0.062	0.0192	0.068	0.028	0.4790	0.9199	0.9311
C7-DC	0.009	0.0048	0.008	0.005	0.4226	0.8836	0.174
C9	0.022	0.0079	0.027	0.008	0. 308	0.4965	0.9558
Dimethyl sulfone.1	9.335	5.9744	9.115	4.909	0.8993	0.9661	0. 1
Ethanol	18.280	9.9662	61.915	79.371	0. 195	0.4894	0.4
Isopropyl alcohol	1.770	0.7533	7.582	12.340	0. 422	0.6176	0.5
Methanol	225.355	274.3413	432.830	372.705	0. 521	0.6230	0.06
Propylene glycol	0.435	0.7604	0.173	0.208	0.1451	0.8709	0.07
Trp	5.260	6.1949	3.776	0.929	0.2960	0.8709	0.05
Myo-inositol	87.670	17.2845	85.362	19.619	0.6952	0.9509	0.1941
Creatine	31.525	4.1801	31.926	5.792	0.8031	0.9657	0.1321
Creatinine	68.340	9.8205	71.960	12.194	0.3077	0.8709	0.7975
Dopamine	0.010	0.0048	0.008	0.006	0.2083	0.8709	0.0003
Histamine	3.270	2.6477	5.450	5.041	0.0950	0.8496	0.098
Putrescine	0.133	0.0481	0.132	0.034	0.9457	0.9761	0.1750
Sarcosine	1.620	0.5175	1.453	0.451	0.2814	0.8709	0.0481

SDMA	0.156	0.0392	0.149	0.041	0.5624	0.9356	0.0322
t4-OH-Pro	0.672	0.3109	0.680	0.217	0.9192	0.9661	0.055
Taurine	8.497	1.7849	9.239	1.952	0.2173	0.8709	0.4459
Pyroglutamic acid	67.730	17.3315	60.091	13.730	0.1306	0.8709	0.7917
Urea	305.365	614.6968	152.363	71.706	0.2758	0.8709	0.2083
Ala	35.520	9.3886	77.585	201.086	0.3559	0.8709	0.3077
Arg	24.070	4.0879	22.770	3.791	0.3036	0.8709	0.3917
Asn	7.359	1.3151	7.012	1.212	0.3917	0.8757	0.3940
Asp	13.047	11.1143	10.552	16.103	0.5851	0.9356	0.0000
Gln	815.450	152.4534	822.100	246.678	0.9189	0.9661	0.0007
Gly	7.555	1.9849	7.933	2.011	0.5527	0.9356	0.9255
His	13.427	6.0346	15.664	2.298	0.1297	0.8709	0.0000
Leu	16.835	6.6081	14.919	4.419	0.2879	0.8709	0.0000
L-Glutamine	50.365	17.1845	43.185	9.744	0.1124	0.8698	0.0040
Lys	39.645	9.7674	38.275	8.878	0.6452	0.9358	0.0056
Met	3.543	0.6141	3.704	0.746	0.4608	0.9159	0.4163
Orn	5.255	1.3536	4.923	1.313	0.4353	0.8984	0.0599
Ser	28.935	5.1213	30.985	6.428	0.2716	0.8709	0.4398
Thr	32.600	7.9827	33.535	5.982	0.6774	0.9484	0.8142
Trp	2.241	0.4498	2.276	0.615	0.8379	0.9661	0.0015
Tyr	9.312	2.0266	9.493	2.568	0.8054	0.9657	0.0120
Val	19.740	7.0516	18.470	5.216	0.5212	0.9356	0.0011
Ile	7.900	3.7155	6.220	3.454	0.1468	0.8709	0.0008
CA	0.010	0.0121	0.006	0.007	0.3104	0.8709	0.0000
CDCA	0.010	0.0123	0.006	0.011	0.3468	0.8709	0.0000
DCA	0.022	0.0147	0.014	0.014	0.1135	0.8698	0.0045
GCA	0.002	0.0041	0.000	0.000	0.0929	0.8496	0.0000
GCDCA	0.020	0.0179	0.009	0.006	0.0153	0.4894	0.0000
GDCA	0.004	0.0049	0.002	0.002	0.1445	0.8709	0.0000
GUDCA	0.002	0.0011	0.002	0.001	0.3580	0.8709	0.0000
HDCA	0.004	0.0055	0.005	0.006	0.8488	0.9661	0.0000
LCA	0.001	0.0028	0.001	0.003	0.9208	0.9661	0.0000
MCA(b)	0.000	0.0002	0.000	0.000	1.0000	1.0000	0.0000

MCA(o)	0.001	0.0014	0.001	0.002	0.6981	0.9509	0.0000
TCA	0.000	0.0015	0.000	0.000	0.1785	0.8709	0.0000
TCDCA	0.002	0.0013	0.002	0.001	0.1982	0.8709	0.0051
TDCA	0.005	0.0024	0.005	0.003	0.9079	0.9661	0.0004
TMCA(a+b)	0.013	0.0006	0.013	0.000	0.7739	0.9657	0.0000
TUDCA	0.004	0.0003	0.004	0.000	0.5602	0.9356	0.0000
UDCA	0.006	0.0052	0.007	0.006	0.3769	0.8709	0.0014
Dimethylamine	1.715	0.6706	1.860	0.923	0.5717	0.9356	0.0000
Acetone	3.625	2.5196	4.619	2.222	0.1936	0.8709	0.0055
Ascorbate	142.965	30.9602	138.140	21.415	0.5699	0.9356	0.0006
cis-Aconitate	2.990	0.8955	2.890	1.021	0.7438	0.9657	0.4611
2-Aminobutyrate	6.700	6.4296	6.900	4.448	0.9095	0.9661	0.0000
2-Hydroxybutyric acid	22.730	7.1268	23.746	6.978	0.6514	0.9364	0.1215
2-Hydroxyisovalerate	3.845	1.2890	3.528	1.153	0.4179	0.8836	0.0884
2-Oxoglutarate	8.525	2.2574	7.735	2.126	0.2617	0.8709	0.5239
3-Hydroxybutyric acid	9.315	3.7445	17.606	11.586	0.929	0.4085	0.2173
3-Hydroxyisobutyrate	11.140	2.5683	10.180	2.112	0.2044	0.8709	0.9849
3-Hydroxyisovalerate	3.600	1.4209	2.950	1.094	0.1133	0.8698	0.0050
3-Hydroxyisovaleric acid	3.675	1.2260	2.964	1.246	0.0769	0.7743	0.0442
Acetic acid	30.405	6.3942	31.001	11.304	0.8385	0.9661	0.0004
Acetoacetate	5.100	1.5990	5.590	1.603	0.3388	0.8709	0.2738
Citric acid	147.975	30.7628	149.108	38.204	0.9183	0.9661	0.9521
Formate	30.155	4.7200	31.304	5.443	0.4800	0.9199	0.2143
L-Lactic acid	1381.950	194.3256	1346.958	184.895	0.5631	0.9356	0.1838
L-Phenylalanine	6.655	5.4009	5.931	1.583	0.5687	0.9356	0.0000
Malonate	8.285	1.9930	8.045	2.043	0.7090	0.9509	0.7850
Pantothenate	9.640	3.1948	10.085	2.781	0.6412	0.9358	0.8148
Pyruvic acid	14.910	9.0009	14.772	11.020	0.9656	0.9902	0.0002
Succinate	11.515	3.0085	10.936	3.567	0.5821	0.9356	0.0142
Acetaminophen	4.075	4.3764	3.215	2.714	0.4597	0.9159	0.0000
Choline	1.030	0.7821	0.830	0.484	0.3370	0.8709	0.0000
lysoPC a C26:1	0.012	0.0122	0.011	0.013	0.8199	0.9661	0.0001
lysoPC a C28:0	0.065	0.0274	0.068	0.028	0.7147	0.9509	0.0289

lysoPC a C28:1	0.032	0.0230	0.025	0.021	0.3440	0.8709	0.0024
PC aa C28:1	0.027	0.0110	0.031	0.010	0.1655	0.8709	0.3275
PC aa C30:0	0.134	0.0284	0.145	0.035	0.2960	0.8709	0.7068
PC aa C32:0	0.818	0.2135	0.899	0.267	0.2953	0.8709	0.0800
PC aa C32:1	0.330	0.0836	0.359	0.097	0.3134	0.8709	0.0185
PC aa C32:2	0.018	0.0119	0.020	0.019	0.6680	0.9434	0.0064
PC aa C34:1	4.576	1.2343	5.053	1.501	0.2792	0.8709	0.3872
PC aa C34:2	1.051	0.1003	1.115	0.104	0.0542	0.6230	0.2006
PC aa C34:4	0.014	0.0048	0.014	0.004	0.6175	0.9356	0.1432
PC aa C36:0	0.179	0.0369	0.175	0.031	0.6626	0.9434	0.1938
PC aa C36:1	0.620	0.1822	0.672	0.178	0.3690	0.8709	0.8543
PC aa C36:2	0.556	0.1799	0.548	0.148	0.8788	0.9661	0.0000
PC aa C36:4	0.480	0.1704	0.463	0.188	0.7614	0.9657	0.0307
PC aa C36:5	0.037	0.0248	0.034	0.008	0.6276	0.9356	0.0000
PC aa C36:6	0.004	0.0022	0.003	0.002	0.3841	0.8709	0.1300
PC aa C38:3	0.223	0.0999	0.223	0.090	0.9960	1.0000	0.4203
PC aa C38:4	0.536	0.1491	0.547	0.186	0.8426	0.9661	0.3089
PC aa C38:5	0.127	0.0527	0.130	0.052	0.8318	0.9661	0.0020
PC aa C38:6	0.202	0.1010	0.187	0.073	0.5839	0.9356	0.0030
PC aa C40:3	0.010	0.0068	0.013	0.006	0.1696	0.8709	0.0004
PC aa C40:4	0.047	0.0178	0.049	0.022	0.7922	0.9657	0.0806
PC aa C40:5	0.035	0.0208	0.041	0.018	0.3509	0.8709	0.5058
PC aa C40:6	0.240	0.0532	0.231	0.043	0.5413	0.9356	0.0415
PC aa C42:2	0.020	0.0083	0.021	0.007	0.7609	0.9657	0.0233
PC ae C30:1	0.006	0.0037	0.006	0.003	0.8578	0.9661	0.5005
PC ae C32:1	0.054	0.0212	0.058	0.024	0.6237	0.9356	0.1751
PC ae C32:2	0.015	0.0075	0.016	0.008	0.9023	0.9661	0.2569
PC ae C34:0	0.028	0.0114	0.030	0.013	0.6411	0.9358	0.0689
PC ae C34:1	0.188	0.0507	0.202	0.063	0.4712	0.9199	0.3594
PC ae C34:2	0.155	0.0647	0.157	0.057	0.9241	0.9661	0.3899
PC ae C34:3	0.021	0.0051	0.019	0.008	0.3563	0.8709	0.1042
PC ae C36:1	0.466	0.0392	0.493	0.048	0.0593	0.6363	0.3073
PC ae C36:2	0.114	0.0138	0.123	0.011	0.0289	0.4965	0.1352

PC ae C36:3	0.030	0.0132	0.032	0.012	0.6224	0.9356	0.0611
PC ae C36:4	0.057	0.0219	0.059	0.021	0.7723	0.9657	0.3573
PC ae C36:5	0.046	0.0174	0.050	0.020	0.4954	0.9273	0.0529
PC ae C38:0	0.018	0.0047	0.019	0.006	0.5657	0.9356	0.0333
PC ae C38:1	0.136	0.0272	0.159	0.021	0.0051	0.4085	0.6541
PC ae C38:2	0.092	0.0162	0.100	0.022	0.2006	0.8709	0.1784
PC ae C38:3	0.091	0.0083	0.099	0.012	0.0213	0.4894	0.0784
PC ae C38:4	0.044	0.0189	0.046	0.020	0.7116	0.9509	0.0020
PC ae C38:5	0.060	0.0207	0.066	0.022	0.3971	0.8757	0.3228
PC ae C38:6	0.021	0.0121	0.019	0.011	0.6226	0.9356	0.2144
PC ae C40:1	0.021	0.0073	0.021	0.008	0.7960	0.9657	0.0002
PC ae C40:2	0.036	0.0053	0.040	0.006	0.0297	0.4965	0.6088
PC ae C40:3	0.044	0.0127	0.046	0.015	0.6028	0.9356	0.0002
PC ae C40:4	0.031	0.0095	0.032	0.013	0.7978	0.9657	0.0296
PC ae C40:6	0.019	0.0078	0.022	0.008	0.3235	0.8709	0.7153
PC ae C44:6	0.009	0.0067	0.012	0.005	0.2405	0.8709	0.0128
H1	11221.789	1777.2594	11859.250	2441.678	0.3594	0.8709	0.9460
Xanthine	1.190	1.4875	0.859	0.229	0.3316	0.8709	0.0000
SM (OH) C14:1	0.042	0.0143	0.039	0.012	0.4129	0.8836	0.1699
SM (OH) C16:1	0.033	0.0157	0.037	0.012	0.3293	0.8709	0.0618
SM (OH) C22:1	0.039	0.0176	0.035	0.008	0.3461	0.8709	0.0000
SM (OH) C22:2	0.044	0.0197	0.043	0.020	0.8993	0.9661	0.0003
SM C16:0	0.836	0.2512	0.807	0.274	0.7274	0.9600	0.4431
SM C16:1	0.098	0.0347	0.089	0.029	0.3755	0.8709	0.1779
SM C18:0	0.781	0.2787	0.825	0.246	0.6018	0.9356	0.2159
SM C18:1	0.208	0.0758	0.209	0.067	0.9947	1.0000	0.2648
SM C20:2	0.001	0.0020	0.001	0.002	0.9425	0.9761	0.0000
SM C24:0	0.145	0.0569	0.123	0.048	0.2120	0.8709	0.1456
SM C24:1	0.464	0.1639	0.453	0.122	0.8075	0.9657	0.8488
SM C26:0	0.005	0.0053	0.006	0.003	0.9719	0.9904	0.0067
SM C26:1	0.008	0.0047	0.006	0.006	0.1300	0.8709	0.0298
Fructose	58.160	16.3454	55.303	19.018	0.6134	0.9356	0.0034
Mannose	27.155	19.4511	23.188	4.729	0.3811	0.8709	0.0000

Glycerol	153.965	40.7197	186.709	59.458	0.0492	0.6230	0.6138
D-Glucose	2299.345	291.7000	2165.120	380.751	0.2184	0.8709	0.0010

Table S8: Pair-wise partial least square discriminant (PLS-DA) model performance for LRKK2 control vs. LRKK2 PD sufferers, sPD controls vs. sPD sufferers and LRKK2 patients vs. sPD sufferers.

**Models**	**Sensitivity**	**Specificity**	**Significance**	**Accuracy**
LRKK2 vs. LRKK2 Control	0.80	0.94	0.032	0.815
sPD vs. sPD Control	0.75	0.76	0.435	0.713
sPD vs. LRKK2	0.74	0.81	0.351	0.977

Table S9: Optimized model parameters for each machine learning algorithm evaluated for prediction of in this study.

Models	Optimized Parameters
logistic_reg	steps=[('preprocess'", ' StandardScaler (copy=True', ' with_mean=True', ' with_std=True))', ('clf'", ' LogisticRegression(C=0.1', ' class_weight=None', ' dual=False', ' fit_intercept=True', ' intercept_scaling=1', ' max_iter=100', " multi_class='warn'", ' n_jobs=None', " penalty='l2'", ' random_state=42', " solver='lbfgs'", ' tol=0.0001', ' verbose=0', ' warm_start=False))
svm_linear	steps=[('preprocess'", ' StandardScaler(copy=True', ' with_mean=True', ' with_std=True))', " ('clf'", ' SVC(C=0.001', ' cache_size=200', ' class_weight=None', ' coef0=0.0', " decision_function_shape='ovr'", ' degree=3', gamma='auto_deprecated'", " kernel='sigmoid'", ' max_iter=-1', ' probability=False', ' random_state=42', ' shrinking=True', ' tol=0.001', ' verbose=False))]
knn	steps=[('preprocess'", ' StandardScaler(copy=True', ' with_mean=True', ' with_std=True))', " ('clf'", " KNeighborsClassifier(algorithm='auto'", ' leaf_size=30', " metric='minkowski'", ' metric_params=None', ' n_jobs=None', ' n_neighbors=3', ' p=2', " weights='uniform'))]
gaussian	steps=[('preprocess'", ' StandardScaler(copy=True', ' with_mean=True', ' with_std=True))', " ('clf'", ' GaussianProcessClassifier(copy_X_train=True', ' kernel=None', ' max_iter_predict=100', " multi_class='one_vs_rest'", ' n_jobs=None', ' n_restarts_optimizer=0', " optimizer='fmin_l_bfgs_b'", ' random_state=None', ' warm_start=False))]
svm kernel	steps=[('preprocess'", ' StandardScaler(copy=True', ' with_mean=True', ' with_std=True))', " ('clf'", ' BernoulliNB(alpha=0.25', ' binarize=0', ' class_prior=None', ' fit_prior=True))])>']
Decision tree	DecisionTreeClassifier(class_weight=None', " criterion='gini'", ' max_depth=2', ' max_features=None', ' max_leaf_nodes=None', ' min_impurity_decrease=0.0', ' min_impurity_split=None', ' min_samples_leaf=1', ' min_samples_split=2', ' min_weight_fraction_leaf=0.0', ' presort=False', ' random_state=42', " splitter='best'))])>"]
ensemble_ada	steps=[('preprocess'", ' StandardScaler(copy=True', ' with_mean=True', ' with_std=True))', " ('clf'", " AdaBoostClassifier(algorithm='SAMME.R'", ' base_estimator=None', ' learning_rate=1.0', ' n_estimators=500', ' random_state=42))])>']
gbm	steps=[('preprocess'", ' StandardScaler(copy=True', ' with_mean=True', ' with_std=True))', " ('clf'", " GradientBoostingClassifier(criterion='friedman_mse'", ' init=None', ' learning_rate=0.1', " loss='deviance'", ' max_depth=1', ' max_features=None', ' max_leaf_nodes=None', ' min_impurity_dec... subsample=1.0', ' tol=0.0001', ' validation_fraction=0.1', ' verbose=0', ' warm_start=False))])>']

Random forest	steps=[('preprocess'", ' StandardScaler(copy=True', ' with_mean=True', ' with_std=True))', " ('clf'", ' RandomForestClassifier(bootstrap=True', ' class_weight=None', " criterion='gini'", ' max_depth=2', " max_features='sqrt'", ' max_leaf_nodes=None', ' min_impurity_decrease=0.0', ' min_impurity_split=None', ' min_samples_leaf=1', ' min_samples_split=2', ' min_weight_fraction_leaf=0.0', ' n_estimators=500', ' n_jobs=None', ' oob_score=False', ' random_state=42', ' verbose=0', ' warm_start=False))]

Table S10: The panel of metabolites as chosen using the RFE feature selection algorithm to generate our predictive models.

Model Ranking	PD_control vs. sPD	LRRK2_control vs. LRRK2	sPD vs. LRRK2
**1**	3-Hydroxybutyric acid	PC ae C38:0	C5
**2**	SM C16:0	Ethanol	PC ae C40:4
**3**	lysoPC a C26:1	Putrescine	lysoPC a C26:1
**4**	PC aa C36:6	PC ae C36:2	2-Hydroxybutyric acid
**5**	Isopropyl alcohol	TCA	SM C16:0

Figure S1: Typical 1D ^{1}H NMR spectrum of CSF from a patient suffering from Parkinson's disease (**a–c**). Metabolites are numbered as follows: DSS, 2-hydroxyisovalerate, 2-hydroxybutryrate pantothenate, leucine, 2-aminobutyrate, isoleucine, 3-hydroxyisovalerate, valine, isopropanol, ethanol, 3-hydroxybutryrate, lactate, alanine, acetate, butyrate, ornithine, acetaminophen, acetone, 3-hydroxyisovalerate, acetoacetate, pyruvate, succinate, 2-oxoglutareta, 3-hydroxyisobutryrate, citrate, glutamine, dimethylamine, creatine, creatinine, cis-aconitate, malonate, dimethyl sulfano, choline, glucose, myo-inositol, histamine, methanol, glycerol, methionine, ascorbate, threonine, mannose, fructose, propylene glycerol, pyroglutamate, urea, tyrosine, phenylalanine, xanthine, formate, histidine, tyrosine.

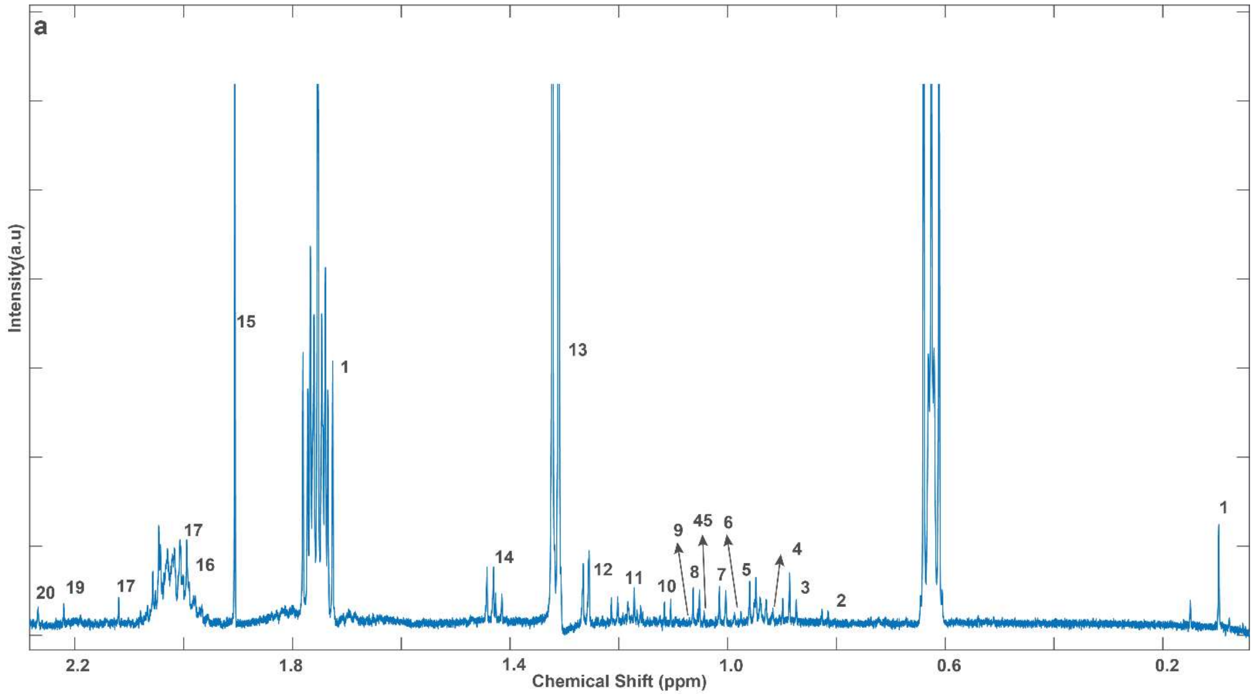

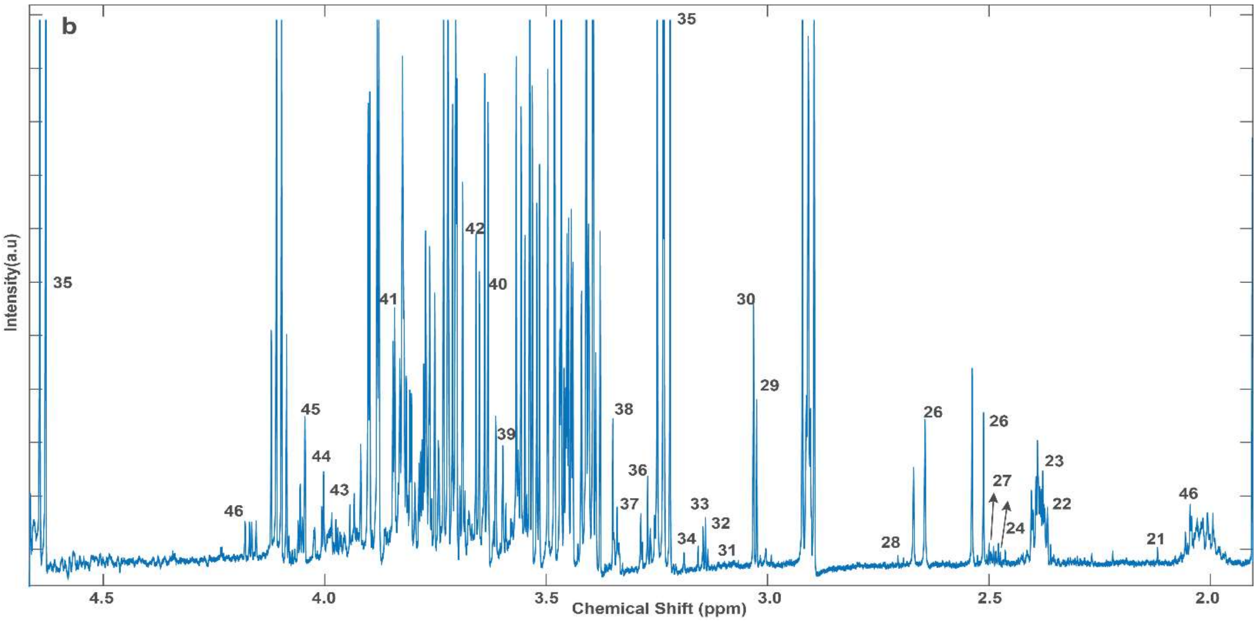

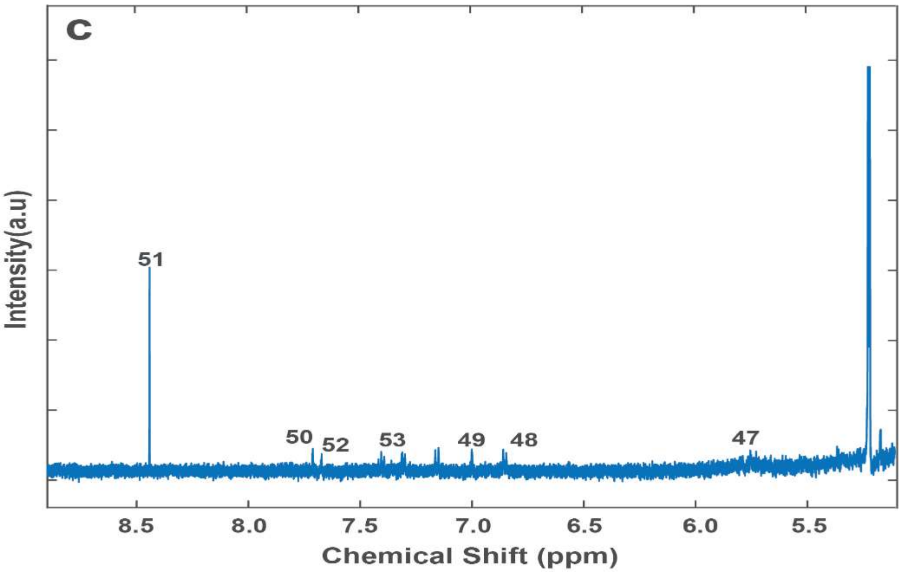
c
51
50
52
53
49
48
47
Intensity(a.u)
8.5
8.0
7.5
7.0
6.5
6.0
5.5
Chemical Shift (ppm)

Figure S2: Investigation of potential outliers for each group by PCA (**a**–**d**) and Q2 vs. T^2Hotelling plots (**e**,**f**).

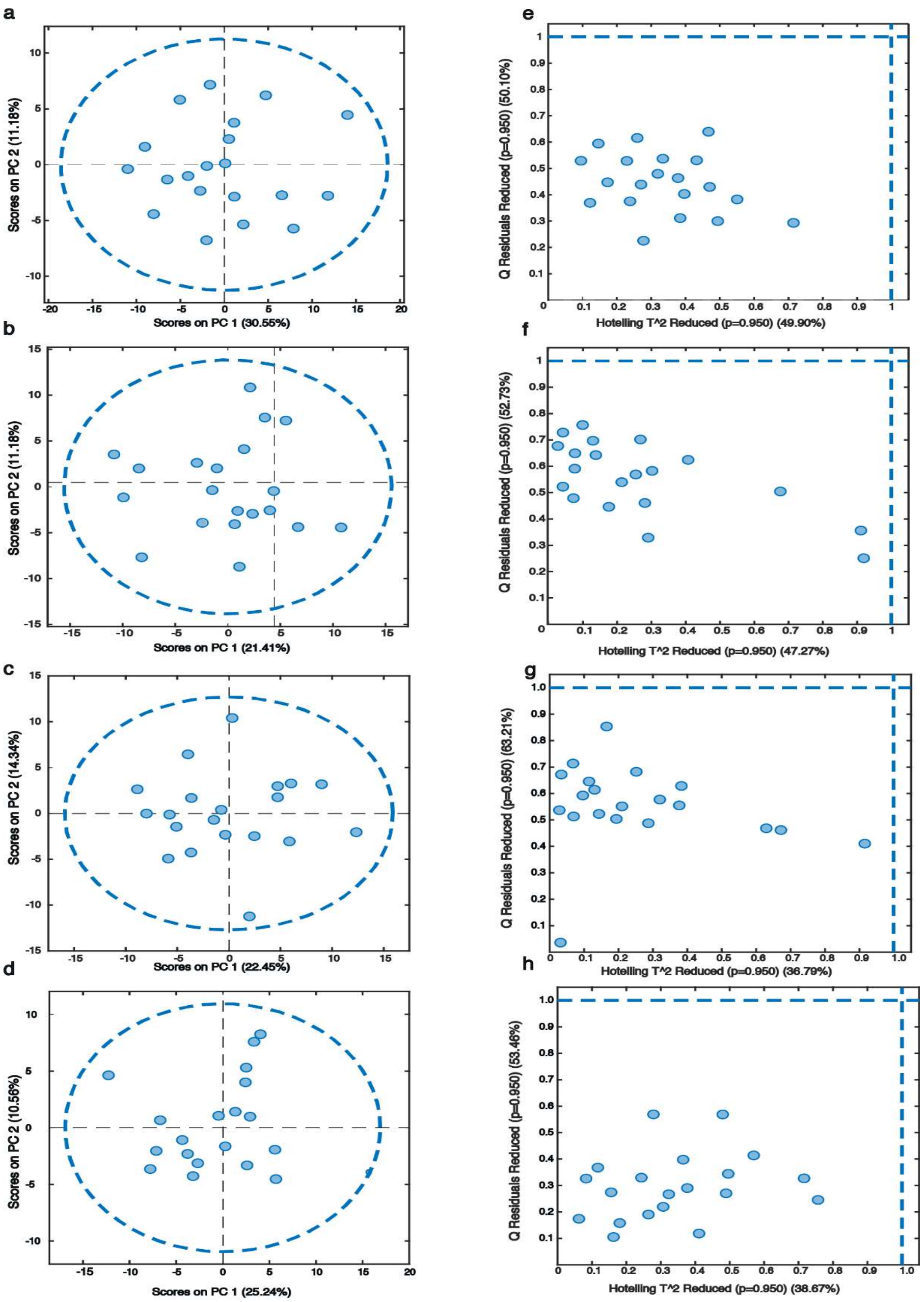

Figure S3: QQ-plots used to determine whether the recorded concentration of metabolomics data is normally distributed and whether the corresponding p-value is inflated.

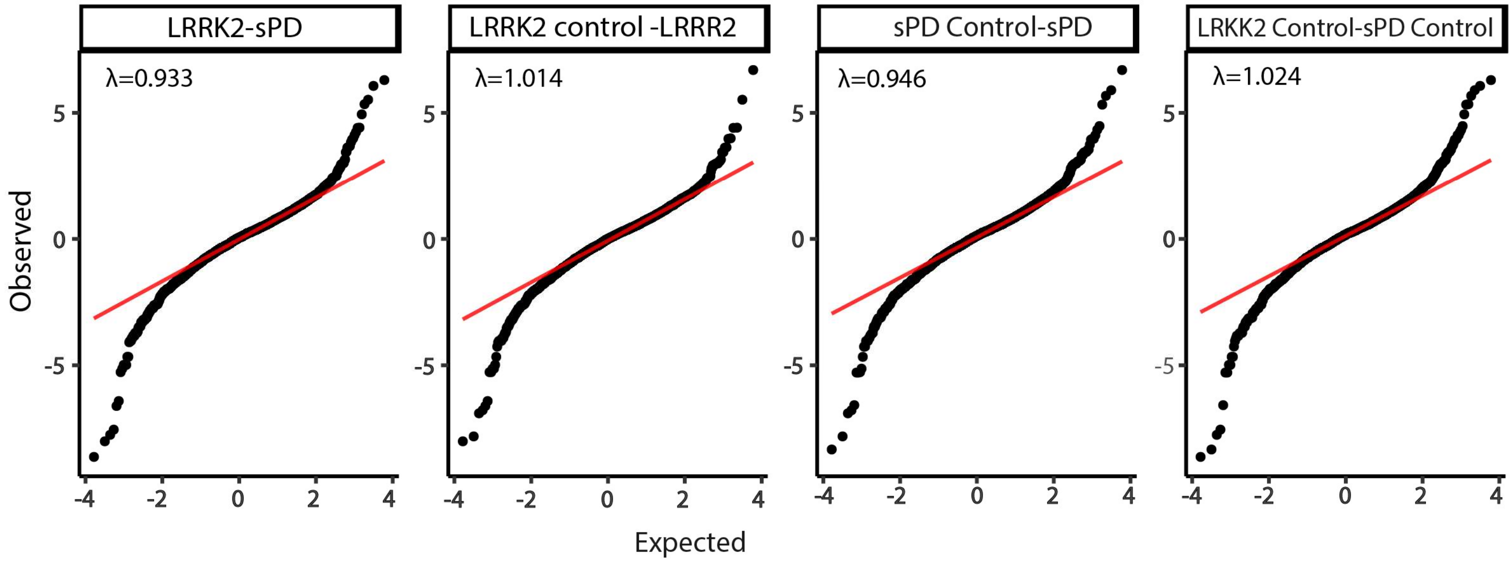

Figure S4: Boxplots of the CSF metabolites found to be at significantly different concentrations across the groups

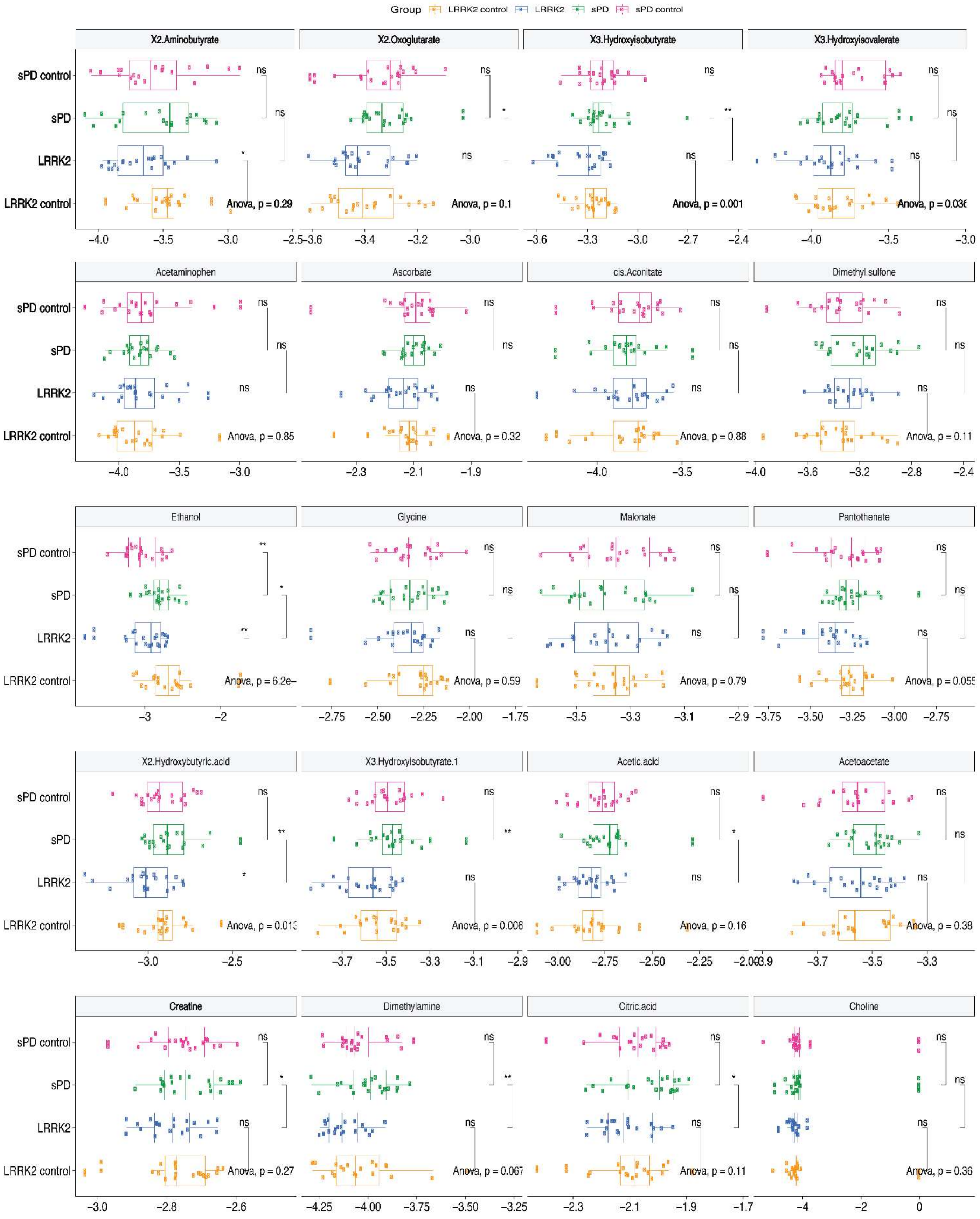

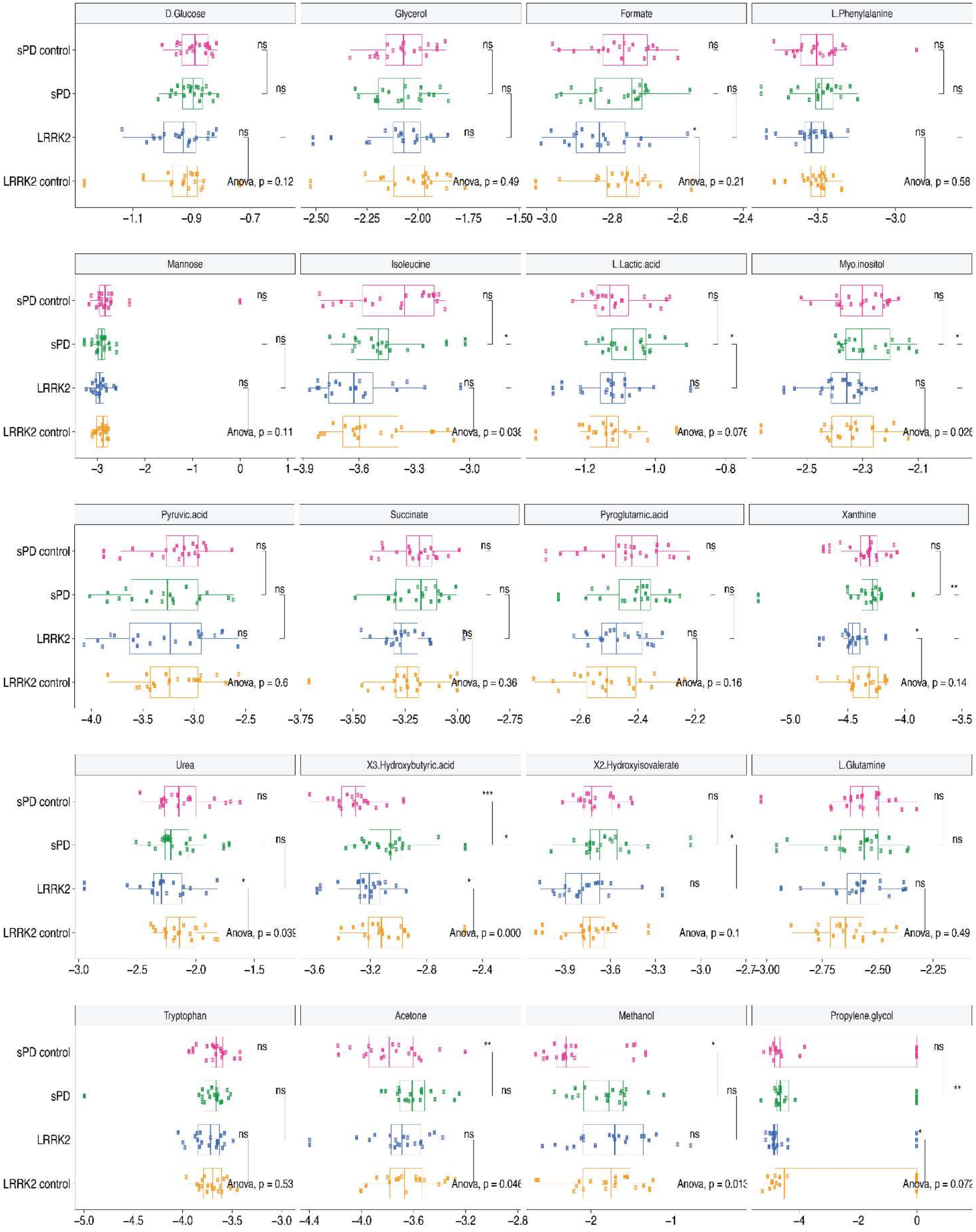

sPD control
sPD
LRRK2
LRRK2 control
D.Glucose
Anova, p = 0.12
Glycerol
Anova, p = 0.49
Formate
Anova, p = 0.21
L.Phenylalanine
Anova, p = 0.58
Mannose
Anova, p = 0.11
Isoleucine
Anova, p = 0.038
L.Lactic.acid
Anova, p = 0.076
Myo.inositol
Anova, p = 0.026
Pyruvic.acid
Anova, p = 0.6
Succinate
Anova, p = 0.36
Pyroglutamic.acid
Anova, p = 0.16
Xanthine
Anova, p = 0.14
Urea
Anova, p = 0.039
X3.Hydroxybutyric.acid
Anova, p = 0.000
X2.Hydroxyisovalerate
Anova, p = 0.1
L.Glutamine
Anova, p = 0.49
Tryptophan
Anova, p = 0.53
Acetone
Anova, p = 0.046
Methanol
Anova, p = 0.013
Propylene.glycol
Anova, p = 0.072

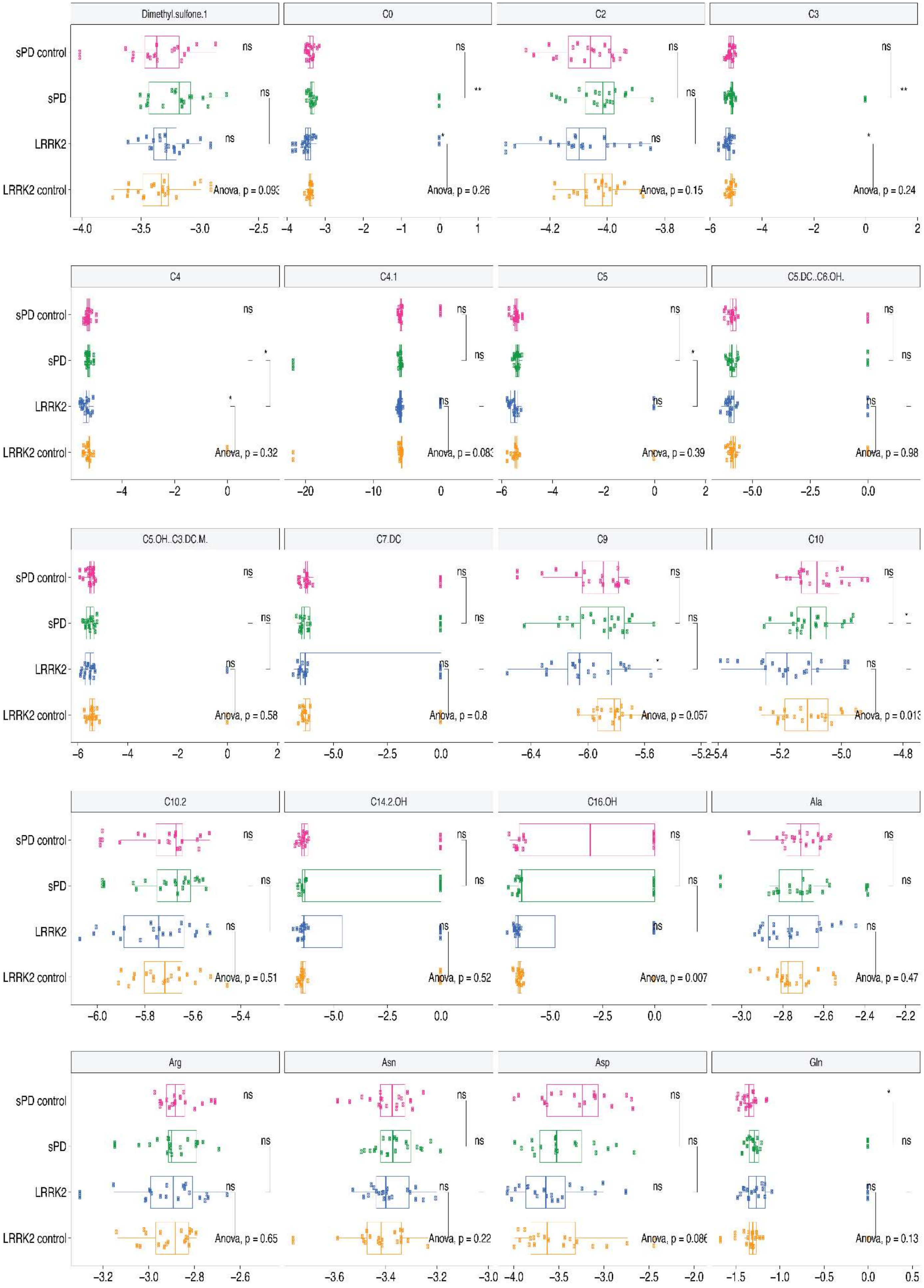

Dimethyl.sulfone.1
C0
C2
C3
C4
C4.1
C5
C5.DC..C6.OH.
C5.OH..C3.DC.M.
C7.DC
C9
C10
C10.2
C14.2.OH
C16.OH
Ala
Arg
Asn
Asp
Gln
sPD control
sPD
LRRK2
LRRK2 control
Anova, p = 0.093
Anova, p = 0.26
Anova, p = 0.15
Anova, p = 0.24
Anova, p = 0.32
Anova, p = 0.083
Anova, p = 0.39
Anova, p = 0.98
Anova, p = 0.58
Anova, p = 0.8
Anova, p = 0.057
Anova, p = 0.013
Anova, p = 0.51
Anova, p = 0.52
Anova, p = 0.007
Anova, p = 0.47
Anova, p = 0.65
Anova, p = 0.22
Anova, p = 0.086
Anova, p = 0.13
ns
*
**

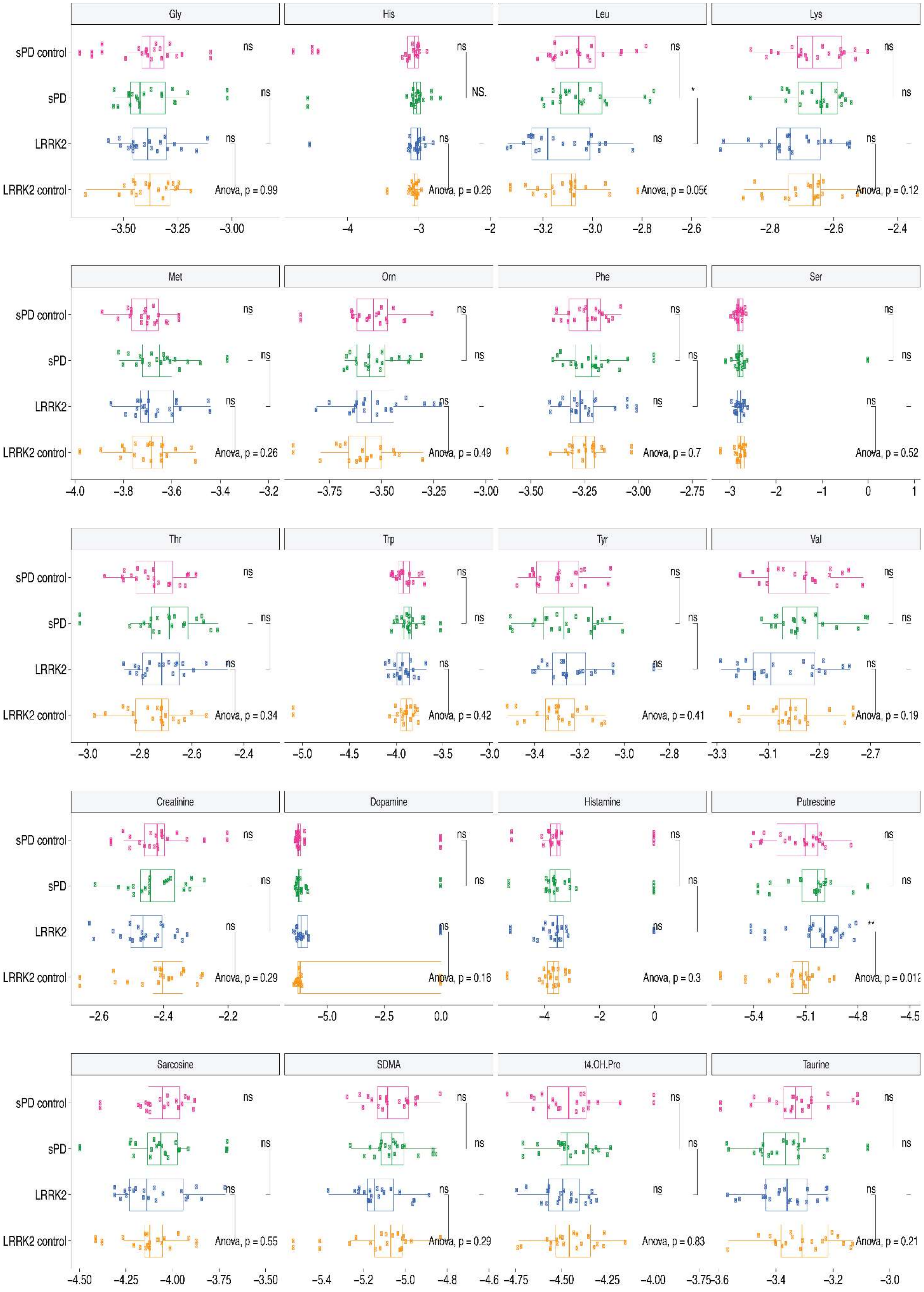
Gly
His
Leu
Lys
sPD control
sPD
LRRK2
LRRK2 control
ns
NS.
*
Anova, p = 0.99
Anova, p = 0.26
Anova, p = 0.056
Anova, p = 0.12
Met
Orn
Phe
Ser
Anova, p = 0.26
Anova, p = 0.49
Anova, p = 0.7
Anova, p = 0.52
Thr
Trp
Tyr
Val
Anova, p = 0.34
Anova, p = 0.42
Anova, p = 0.41
Anova, p = 0.19
Creatinine
Dopamine
Histamine
Putrescine
**
Anova, p = 0.29
Anova, p = 0.16
Anova, p = 0.3
Anova, p = 0.012
Sarcosine
SDMA
t4.OH.Pro
Taurine
Anova, p = 0.55
Anova, p = 0.29
Anova, p = 0.83
Anova, p = 0.21

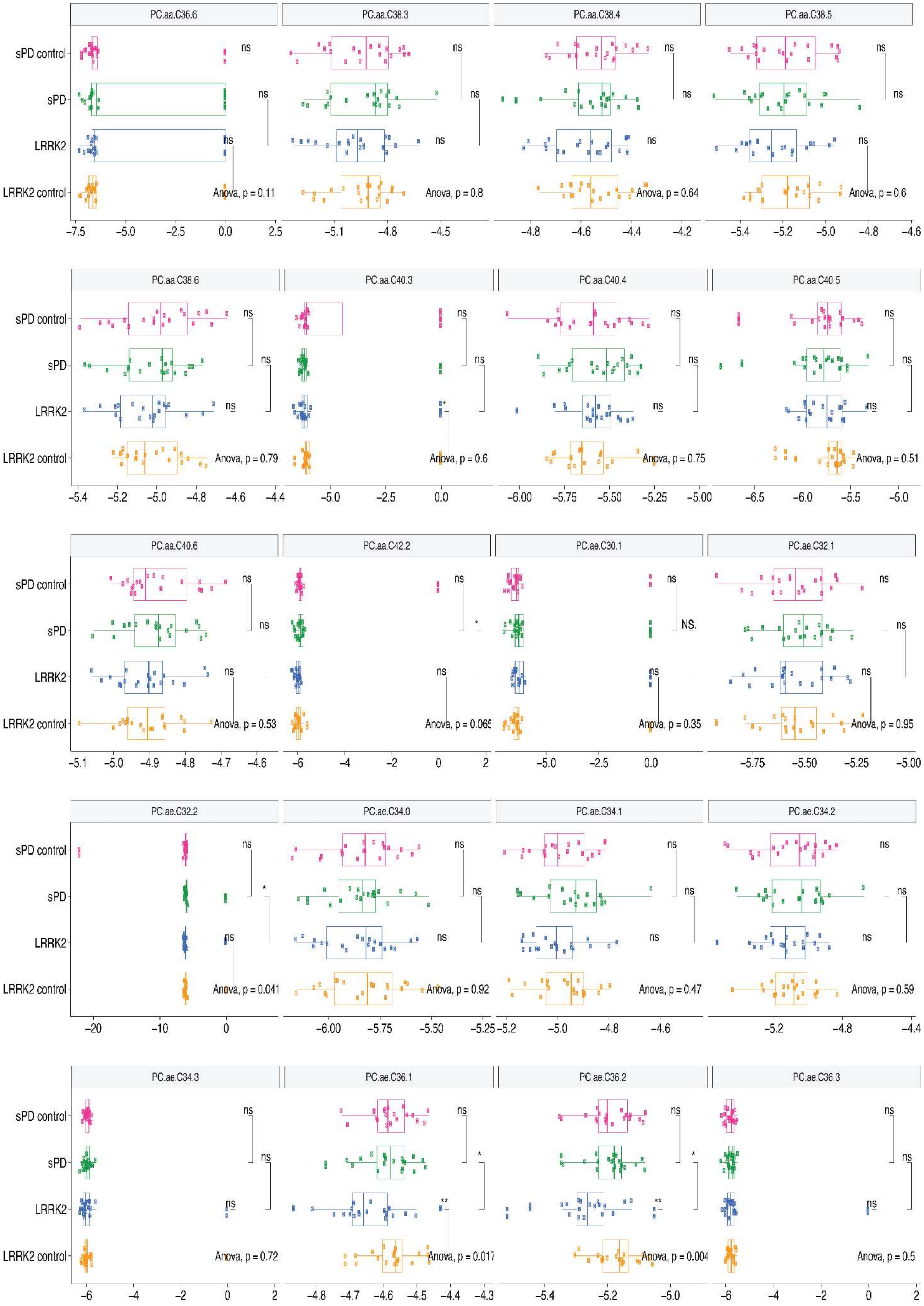
PC.aa.C36.6
PC.aa.C38.3
PC.aa.C38.4
PC.aa.C38.5
PC.aa.C38.6
PC.aa.C40.3
PC.aa.C40.4
PC.aa.C40.5
PC.aa.C40.6
PC.aa.C42.2
PC.ae.C30.1
PC.ae.C32.1
PC.ae.C32.2
PC.ae.C34.0
PC.ae.C34.1
PC.ae.C34.2
PC.ae.C34.3
PC.ae.C36.1
PC.ae.C36.2
PC.ae.C36.3
sPD control
sPD
LRRK2
LRRK2 control
Anova, p = 0.11
Anova, p = 0.8
Anova, p = 0.64
Anova, p = 0.6
Anova, p = 0.79
Anova, p = 0.6
Anova, p = 0.75
Anova, p = 0.51
Anova, p = 0.53
Anova, p = 0.065
Anova, p = 0.35
Anova, p = 0.95
Anova, p = 0.041
Anova, p = 0.92
Anova, p = 0.47
Anova, p = 0.59
Anova, p = 0.72
Anova, p = 0.017
Anova, p = 0.004
Anova, p = 0.5
ns
NS.
*
**

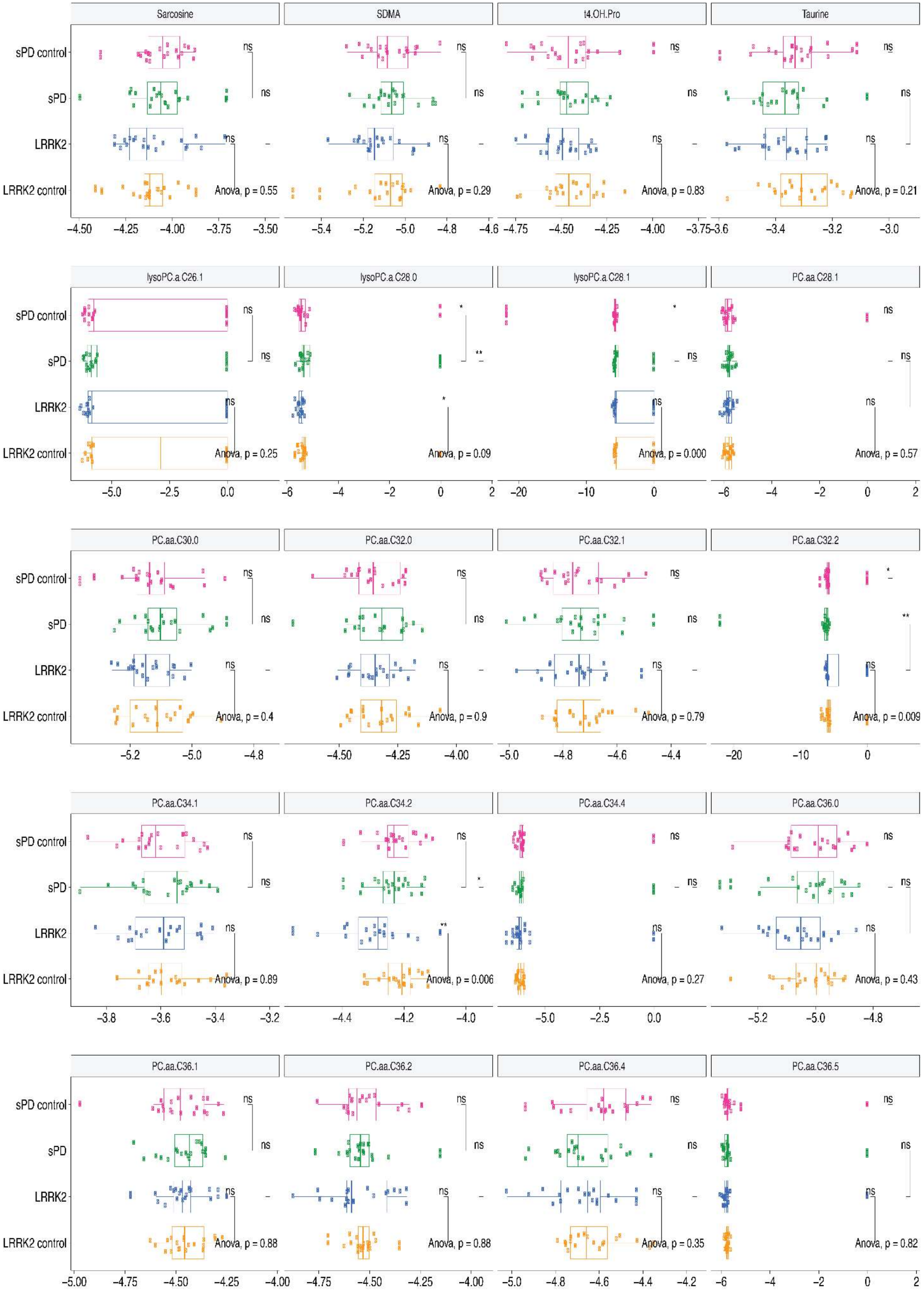
Sarcosine
SDMA
t4.OH.Pro
Taurine
sPD control
sPD
LRRK2
LRRK2 control
Anova, p = 0.55
Anova, p = 0.29
Anova, p = 0.83
Anova, p = 0.21
lysoPC.a.C26.1
lysoPC.a.C28.0
lysoPC.a.C28.1
PC.aa.C28.1
Anova, p = 0.25
Anova, p = 0.09
Anova, p = 0.000
Anova, p = 0.57
PC.aa.C30.0
PC.aa.C32.0
PC.aa.C32.1
PC.aa.C32.2
Anova, p = 0.4
Anova, p = 0.9
Anova, p = 0.79
Anova, p = 0.009
PC.aa.C34.1
PC.aa.C34.2
PC.aa.C34.4
PC.aa.C36.0
Anova, p = 0.89
Anova, p = 0.006
Anova, p = 0.27
Anova, p = 0.43
PC.aa.C36.1
PC.aa.C36.2
PC.aa.C36.4
PC.aa.C36.5
Anova, p = 0.88
Anova, p = 0.88
Anova, p = 0.35
Anova, p = 0.82

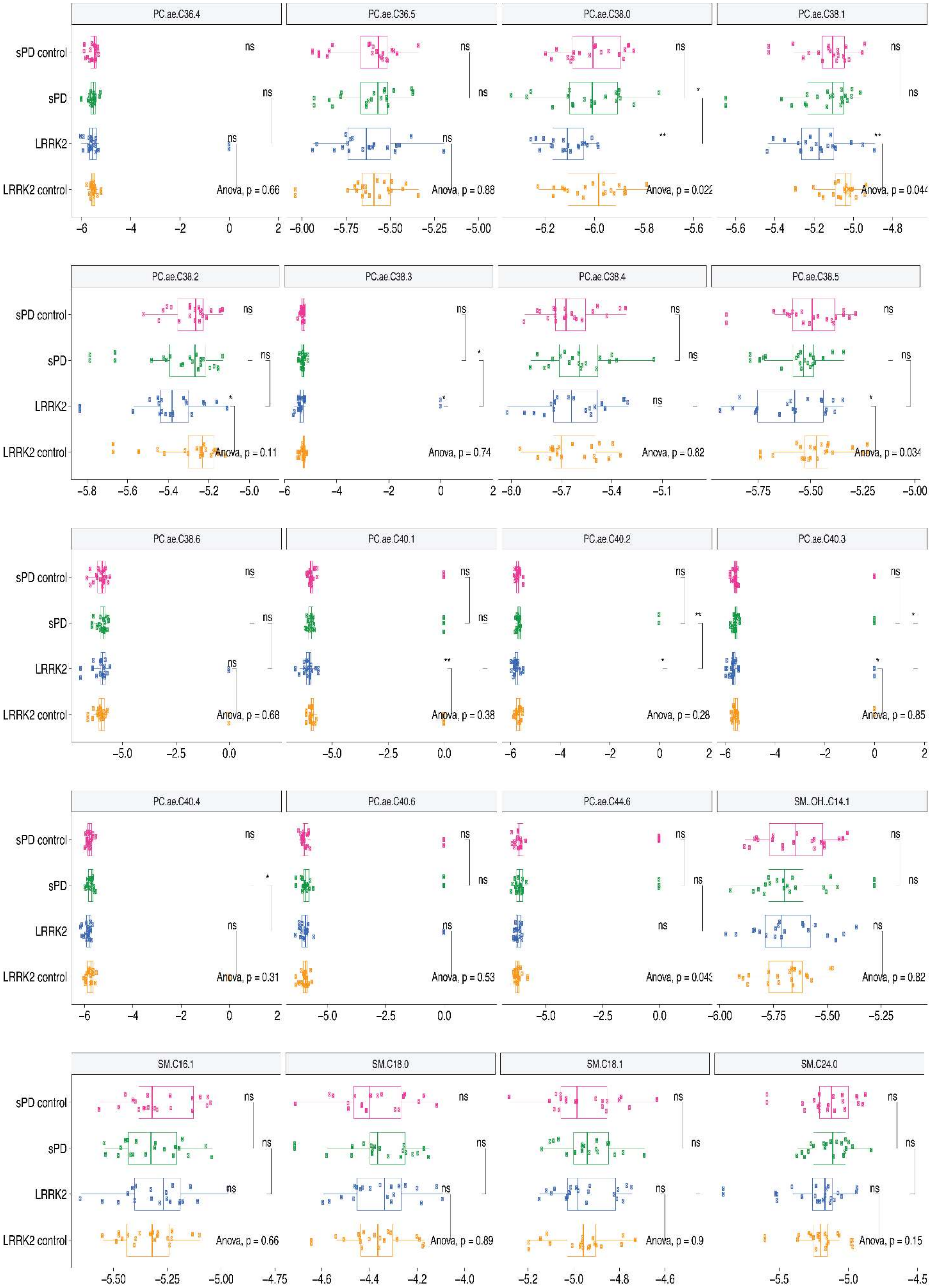
PC.ae.C36.4
PC.ae.C36.5
PC.ae.C38.0
PC.ae.C38.1
sPD control
sPD
LRRK2
LRRK2 control
Anova, p = 0.66
Anova, p = 0.88
Anova, p = 0.022
Anova, p = 0.044
PC.ae.C38.2
PC.ae.C38.3
PC.ae.C38.4
PC.ae.C38.5
Anova, p = 0.11
Anova, p = 0.74
Anova, p = 0.82
Anova, p = 0.034
PC.ae.C38.6
PC.ae.C40.1
PC.ae.C40.2
PC.ae.C40.3
Anova, p = 0.68
Anova, p = 0.38
Anova, p = 0.28
Anova, p = 0.85
PC.ae.C40.4
PC.ae.C40.6
PC.ae.C44.6
SM..OH..C14.1
Anova, p = 0.31
Anova, p = 0.53
Anova, p = 0.043
Anova, p = 0.82
SM.C16.1
SM.C18.0
SM.C18.1
SM.C24.0
Anova, p = 0.66
Anova, p = 0.89
Anova, p = 0.9
Anova, p = 0.15

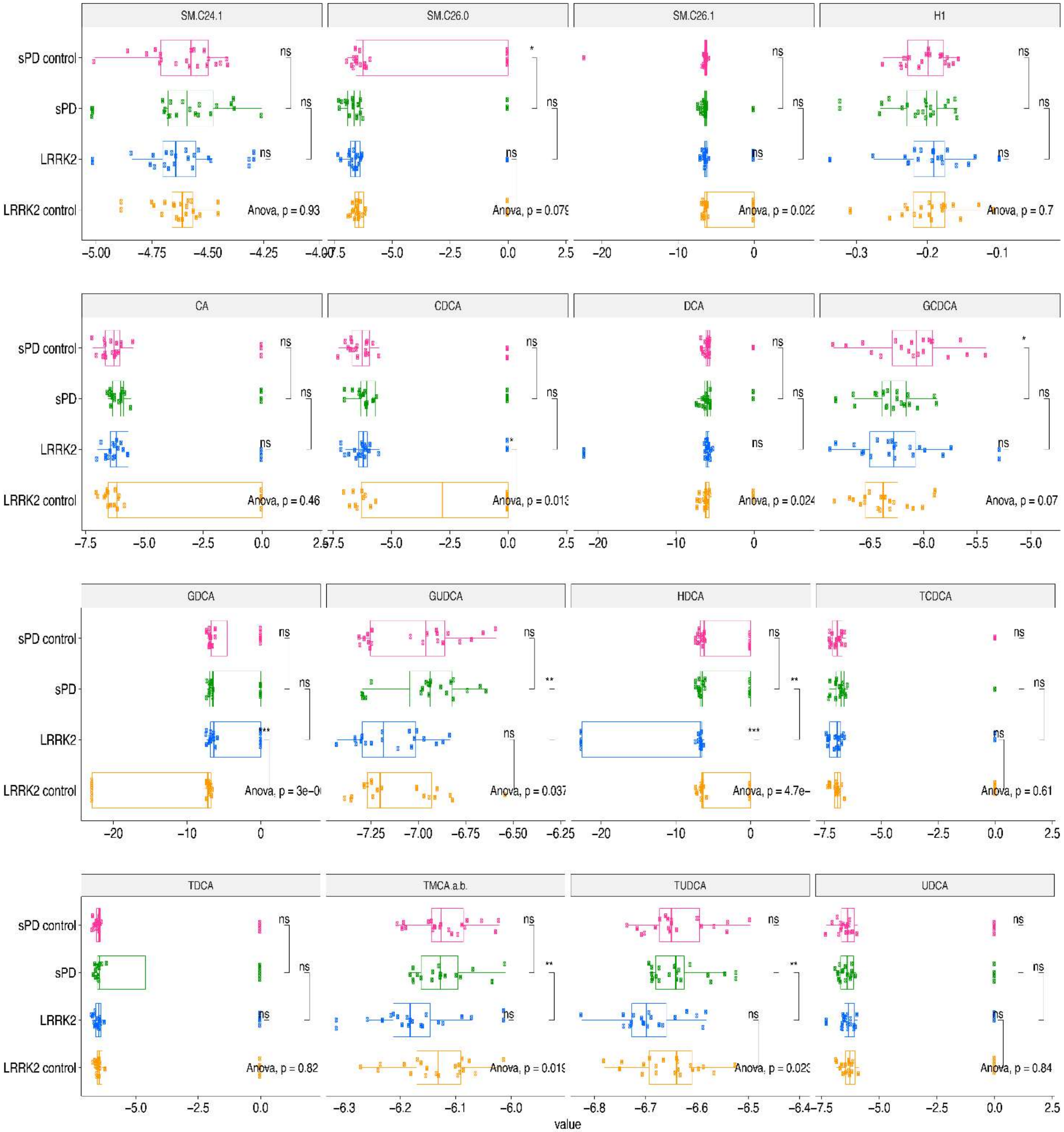

ns: No significant difference in the average concentration values of certain metabolite between the two groups was observed.

**: A significant difference in the average concentration values of certain metabolite between sPD and sPD control groups was observed.*

***: A significant difference in the average concentration values of certain metabolite between LRKK2 and LRKK2 control groups was observed*

****: A significant difference in the average concentration values of certain metabolite between LRKK2 and sPD groups was observed*

Figure S5: AUROC plot for the top three ML-based predictive models for the classification of LRRK2 PD as compared to the corresponding controls.

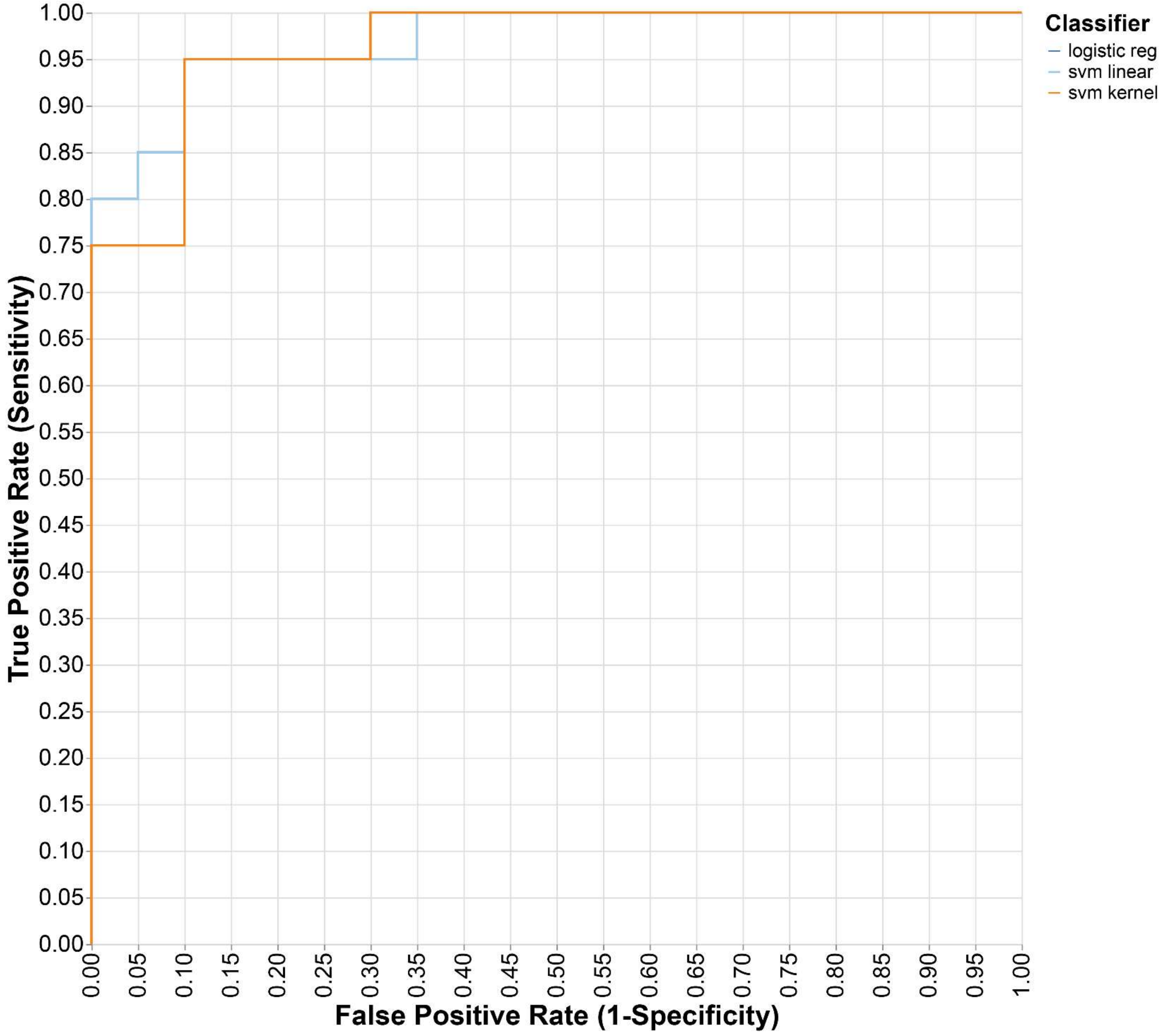

Figure S6: AUROC plot for top three ML-based predictive models for the classification of sPD as compared to the corresponding controls.

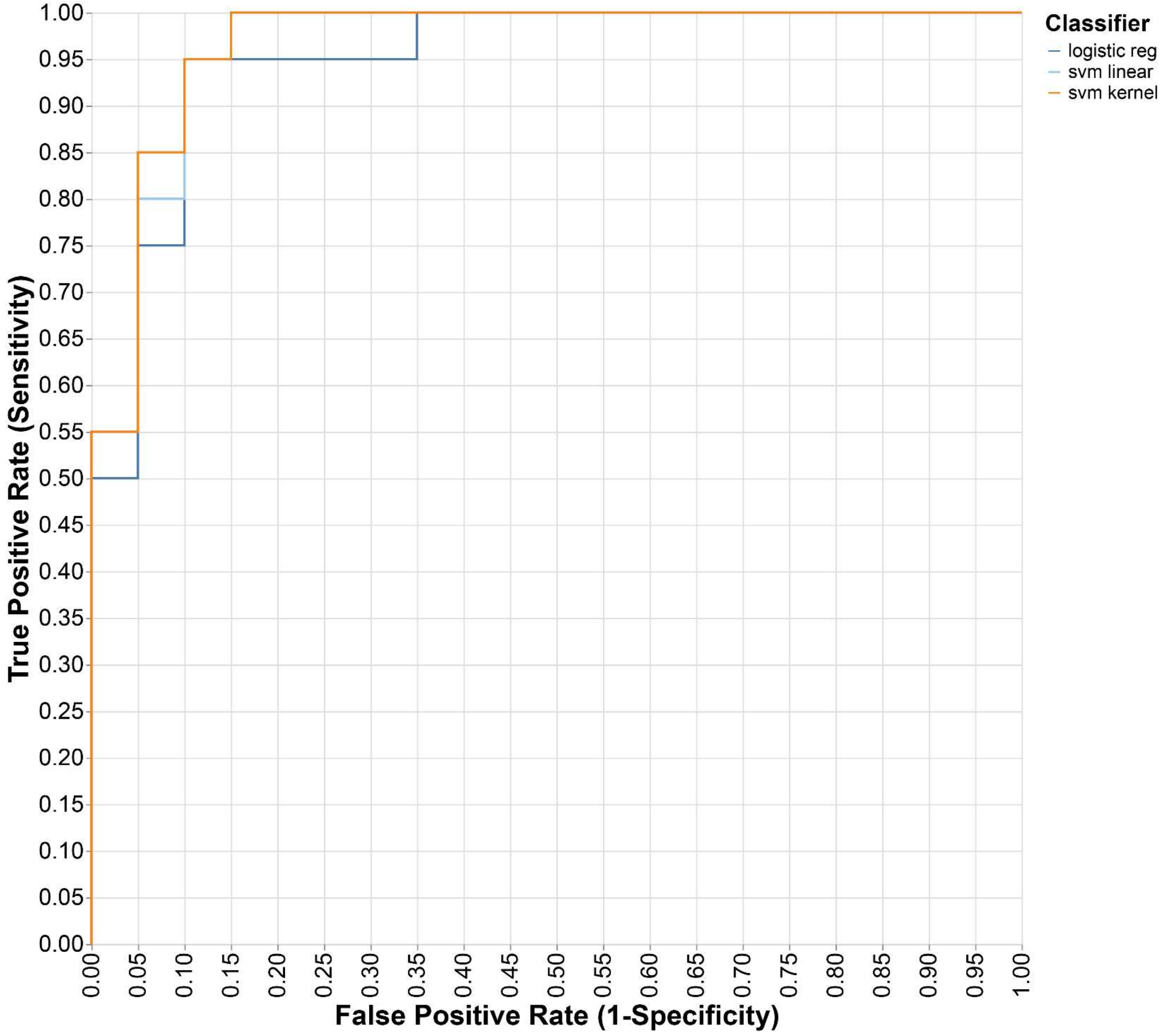

Figure S7: AUROC plot for top three ML-based predictive models for the classification of sPD as compared to LRRK2 PD.

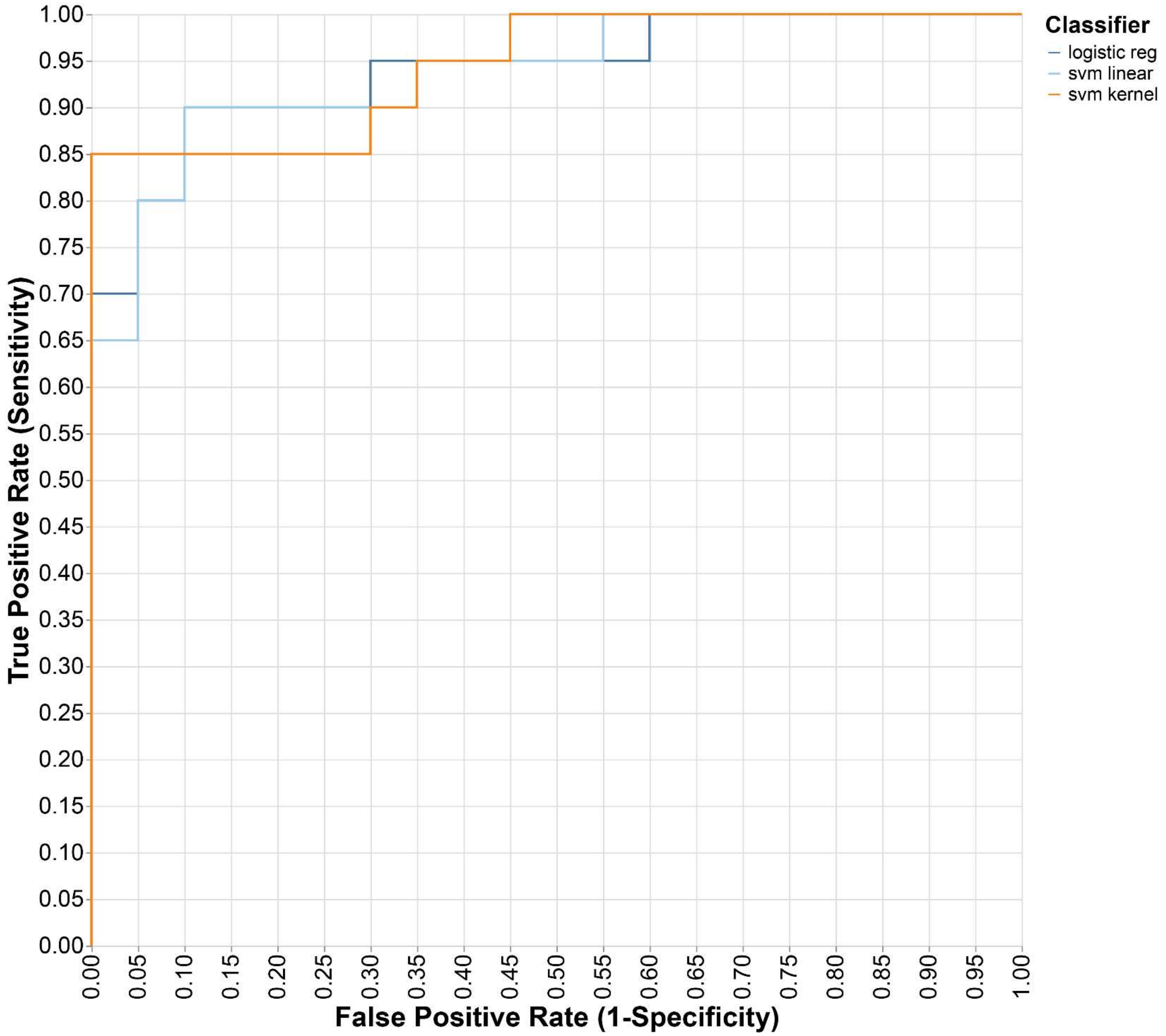

Supplement: Supplementary file 1 [file cells-09-02394-s001.pdf]
